# Performance valuation of onion (*Allium cepa* L.) genotypes under different levels of salinity for the development of cultivars suitable for saline regions

**DOI:** 10.3389/fpls.2023.1154051

**Published:** 2023-03-31

**Authors:** Md. Ashraful Alam, Md. Atikur Rahman, Md. Marufur Rahman, Md. Mahmudul Hasan, Shamsun Naher, Abu Hena Faisal Fahim, Md. Abdul Mottalib, Srabanti Roy, Md. Rafiqul Islam, Shailendra Nath Mozumder, Amnah Mohammed Alsuhaibani, Ahmed Gaber, Akbar Hossain

**Affiliations:** ^1^ Plant Breeding Division, Spices Research Centre, Bangladesh Agricultural Research Institute, Bogura, Bangladesh; ^2^ Division of Soil Science, Spices Research Centre, Bangladesh Agricultural Research Institute, Bogura, Bangladesh; ^3^ Regional Station, Bangladesh Institute of Research and Training on Applied Nutrition, Rangpur, Bangladesh; ^4^ Division of Horticulture, Spices Research Centre, Bangladesh Agricultural Research Institute, Bogura, Bangladesh; ^5^ Division of Agronomy, Spices Research Centre, Bangladesh Agricultural Research Institute, Bogura, Bangladesh; ^6^ Division of Agricultural Engineering, Spices Research Centre, Bangladesh Agricultural Research Institute, Bogura, Bangladesh; ^7^ Department of Agricultural Chemistry, Bangladesh Agricultural University, Mymensingh, Bangladesh; ^8^ Division of Agronomy, Regional Agricultural Research Station, Bangladesh Agricultural Research Institute (BARI), Ishwardi, Pabna, Bangladesh; ^9^ Department of Physical Sport Science, College of Education, Princess Nourah bint Abdulrahman University, Riyadh, Saudi Arabia; ^10^ Department of Biology, College of Science, Taif University, Taif, Saudi Arabia; ^11^ Division of Soil Science, Bangladesh Wheat and Maize Research Institute, Dinajpur, Bangladesh

**Keywords:** salinity, onion, STI, stress, bulb growth, germination

## Abstract

Abiotic stress, especially salt stress, is one of the major barriers to crop production worldwide. Crops like onion that belong to the glycophytic group are more sensitive to salinity stress. A huge study regarding the influence of salinity stress on the growth and development of crops has already been done and is still ongoing. One of the major targets of the research is to develop genotypes that have enhanced performance under stress environments. The world needs more of these types of genotypes to combat the ever-growing salt-stressed soils. Therefore, a number of germplasm were studied during the 2019–2020 and 2020–2021 seasons under different salt concentrations to identify tolerant genotypes as well as to study the plants’ responses at different growth stages against elevated salinity levels. A 2-year study was conducted where germination potential was evaluated in the first year and carried out in petri dish culture of seeds, followed by plastic pot culture for plant establishment and bulb development evaluation during the second year. Four different saline water solutions having different salt concentrations (0, 8, 10, and 12 dS m^−1^) were applied to the petri dishes and pots as the source of water for plants in both seasons. Results indicated that a significant reduction in plants’ performance occurs under higher salinity levels. Salt concentration had an adverse impact on germination, leaf development and growth, the height of plants, bulb size and shape, and the bulb weight of onion. All the growth phases of onion are sensitive to elevated concentrations. Variable performances were observed in the genotypes under stress conditions, and a few genotypes (Ac Bog 409, Ac Bog 414, Ac Bog 424, Ac Bog 430, Ac Bog 417, Ac Bog 419, Ac Bog 420, Ac Bog 422, and Ac Bog 425) having some sort of tolerance to salt stress were identified, which might be recommended for mass production. Tolerance indices could successfully be applied in selecting the salt-tolerant genotypes. Thus, the present findings and the identified genotypes could be further utilized in salt stress improvement research on onion.

## 1 Introduction

Onion (*Allium cepa* L.) is one of the most important vegetables and spice crops. It is one of the most popular vegetables around the globe, carrying a pungent flavor and often used as a condiment to prepare multiple delicious cuisines in every corner of the world. It has important medicinal properties to combat several diseases especially blood pressure and heart disease. Two-thirds of the total onion production come from Asia, among which India and China hold the major share (FAOSTAT, [[NoYear]]). Bangladesh ranks third in the list in terms of production (Star Business Report, 2022). Bangladesh produced 19.54 lakh M tons of onion bulbs from 1.85 lakh ha of land in 2020, with an average yield of 10.55 t/ha (FAOSTAT, [[NoYear]]), which is very low compared to other countries. Onion ranks the highest among the spice crops in Bangladesh based on production and generally grows all over the country mostly in the winter season. It is an important ingredient in many food preparations and is mostly used as a spice rather than as a vegetable in different daily dishes. Although the country is producing a lot of onion, it is still has a huge shortage and, thus, has to import a large amount from abroad to meet the domestic demand (BBS, 2019).

To feed the ever-increasing population, food production by irrigation is common in arid and semiarid regions, resulting in 20% to 50% of the land being affected by salinity called secondary salinization, bringing unprecedented agricultural losses over time (Pitman and Läuchli, 2006). A similar statement was also concluded in a prediction that highlights that approximately 50% of today’s arable land worldwide would be lost from agricultural use due to the worse effect of salinity by 2050 (Wang et al., 2003). It is estimated that there is a loss of more than US$12 billion per year worldwide due to salinity-induced agricultural input losses (Shabala, 2013).

Soil salinity is one of the harsh outputs of global climate change and has an immense impact on arable land, especially coastal agricultural land (Qadir et al., 2014; Rahman et al., 2018). Soil degradation by salinization is one of the consequences of climate change caused by natural and anthropogenic activities (Yeo, 1998). An elevated salinity level adversely affects the morphology, physiology, and yield of a crop and is similar to the case of onion production as well (Shoaib et al., 2018; Regessa et al., 2022; Sanwal et al., 2022; Venâncio et al., 2022). Germination and emergence become difficult (Khan, 2003; Regessa et al., 2010; Hanci and Cebeci, 2015; Ullah and Bano, 2019) and subsequent yield reduction occurs (Chinnusamy et al., 2005) for glycophytes under saline-affected soils (Hanci et al., 2016), although they have a different threshold level of salinity, such as the onion, which is very sensitive to salinity beyond 1.2 dS m^−1^ (Maas and Hoffman, 1977).

In Bangladesh, onion is being grown all over the country, but production is hampered in saline-prone areas around the coastal belt as it is a glycophytic crop. A total of 1.06 million ha of land area (32% of the total coastal and offshore land) in the country is affected by different degrees of salinity (Ahsan and Bhuiyan, 2010; SRDI, 2010; Parvin et al., 2017). Soil salinity was classified by Soil Research Development Institute (SRDI) (2010) as non-saline (2.0–4.0 dS m^−1^), very slightly saline (4.1–8.0 dS m^−1^), moderately saline (8.1–12.0 dS m^−1^), strongly saline (12.1–16.0 dS m^−1^), and very strongly saline (>16 dS m^−1^), which occupied approximately 0.328 (31%), 0.274 (26%), 0.190 (18%), 0.162 (15%), and 0.102 (10%) m ha of land, respectively (Ahsan and Bhuiyan, 2010; SRDI, 2010). During the growing season, salinity level varies between 6 and 12 dS m^−1^, which reaches up to 20 dS m^−1^ in extreme cases (SRDI, 2010). Thus, the cropping intensity is low in the coastal areas compared to the national average. A substantial amount of land has always remained fallow in coastal areas during the winter season after Aman rice (wet season rice) cultivation due to salinity problems. Onion has the potential as a cash crop to fit in this area to increase cropping intensity and save foreign currency by reducing the import of the crop, if cultivated after harvesting T. Aman rice. Moreover, onion cultivation in the mainland has almost plateaued, thus requiring a higher production to meet the shortage. Public research institutes in the country developed several varieties of onion suitable for both winter and rainy seasons, mainly on the mainland. However, those also suffer from salinity stress when cultivated in coastal areas. Therefore, the yield of onion in this area is very low compared to other parts of the country. Hence, genotypes suitable for cultivation under low to moderate levels of salinity level are a crying need for this zone. The potential genotype tolerant to salt stress will increase the total onion production in the country and improve the socio-economic condition of farmers. It will also play a role in increasing the onion cultivation area in the coastal belt. By keeping the above view, the present study was hypothesized as an attempt to evaluate and identify suitable genotypes that have a tolerance to moderate salinity (8–12 dS m^−1^) levels to increase onion production in saline-prone regions.

## 2 Materials and methods

### 2.1 Location of the current study

The present study was carried out at the Spices Research Centre, BARI, Bogura during two consecutive winter seasons (*Rabi*): 2019 and 2020. The details of prevailing weather conditions during the pot experiment are given in **Table S1**.

### 2.2 Properties of soil used in the study

Physicochemical analysis of initial soil under field conditions was carried out at the central soil science laboratory of BARI. The soil employed in the study had a sandy loam texture and was slightly neutral in nature in response, with a field capacity of 29.6% and a pH of 6.0, and organic matter percentage was low (1.33), having an average EC of 2.06 dS m^−1^. Total nitrogen (0.07%) was very low, available phosphorus (40.77 µg/g soil) was very high, exchangeable potassium was low (0.15 meq/100 g soil), and available sulfur was low (10.57 µg/g soil). Available iron (85.91 µg/g soil) was very high, available zinc (1.63 µg/g soil) was optimum, available boron (0.2 µg/g soil) was low, available manganese (18.21 µg/g soil) was very high, available copper was very high (2.31 µg/g soil), exchangeable calcium (4.70 meq/100 g soil) was optimum, and exchangeable magnesium (1.59 meq/100 g soil) was high.

### 2.3 Genotypes used in the study

A set of 25 onion genotypes was included in the current study; details of the studied genotypes are presented in **Table S1** (Khan et al., 2022). A local cultivar BARI Piaz-4 released from a public research institute (BARI) was incorporated as a check cultivar.

### 2.4 Treatments and design

Four different salinity levels were applied in the present experiment (0, 8, 10, and 12 dS m^−1^) for both petri dish and pot experiments. The saline solutions were prepared using normal NaCl salt following the method recommended by Yaron and Mokady (1962). In the first year, the observation was done in petri dishes by arranging all treatments in a completely randomized design (CRD) and repeated three times. In the second-year experiment, all genotypes were accommodated in small-sized pots placed in the field and also laid out in a CRD by repeating all treatments two times.

### 2.5 Experimental plan

In the first season, all the genotypes were tested for their ability to germinate and subsequent plumule development under different salinity treatments. Autoclaved petri dishes were used, where blotting papers (Whatman no. 1) were placed. Then, seeds of the studied genotypes were placed in different petri dishes. Four levels of saline water were applied for each genotype to indulge the germination process. Spraying of saline water as per treatments was applied every other day. The percentage of germination and growth repression was observed after 7 days.

During the second season, the genotypes under study were accommodated in small-sized pots placed in the field. At first, seeds of the different genotypes were sown on a well-prepared seed bed to grow seedlings. The seedlings were then transplanted into the pot and kept in the field at 35 days after sowing. Uniform sandy loam soil was collected from AEZ-4 and then recommended doses of compost and chemical fertilizers were incorporated for this experiment. The experimental pot was fabricated by biodegradable plastic materials having an internal volume of 500 ml. Each pot was filled with 400 g of soil mixture. A 15 cm × 10 cm spacing was maintained from row to row and hill to hill while arranging pots in the field. Starting from transplanting, irrigations were applied with particular saline solutions to reach the field capacity of soil as per aforesaid treatments. Other intercultural practices were followed as and when required, in which timely irrigation was provided to ensure moisture availability and plant protection measures were taken to repel pest and disease infestation.

### 2.6 Observations recorded

Various morpho-physiological trait observations were recorded using a standard protocol for onion phenotyping. The germination percentage was recorded after 7 days of seed placement on petri dishes. Subsequent growth repression (further growth and development arrested despite seed germination) was observed after 14 days of seed placement on each petri dish. The number of plants with dried leaves, number of green plants, maximum leaf length (MLL), and number of total leaves were counted on each pot basis. Individual bulb weight (IBW), bulb length (BL), and bulb diameter (BD) were recorded at harvest. Data on soil salinity level were observed eight times during the entire crop cycle from the seedling to the harvesting stage with an EC meter (model: HI 993310) after 15 days of each irrigation as indicated by Slavich and Petterson (1993) (Slavich and Petterson, 1993).

### 2.7 Statistical analysis

All the observed data were subjected to statistical analysis following the standard formulas. The Data Analysis tool of the Microsoft Excel program was used to estimate the statistical parameters. CV% was estimated based on output from the analyzed results. Shoot tolerance index (ShTI), stress tolerance index (STI), and percent yield reduction (PYR) were calculated on the MS Excel program following the formula given below (Fischer and Maurer, 1978; Choukan et al., 2006; Takahashi et al., 2015; Zafar et al., 2015; Guellim et al., 2019).

The ShTI was estimated according to the following equation:


…………………… [i] $$\text{ShTI }=\frac{\text{MLL at different dS}/\text{m}}{\text{MLL at }0\text{ dS}/\text{m}}\;\times \;100$$

The STI was estimated according to the following equation:


…………………… [ii] $$\text{STI }=\frac{\text{IBW at }0\text{ dS}/\text{m x IBW at different dS}/\text{m}}{\left(\text{Grand mean of IBW}\right)2}$$

The PYR was estimated according to the following equation:


…………………… [iii] $$\text{PYR }=\frac{\text{IBW at }0\text{ dS}/\text{m}-\text{ IBW at different dS}/\text{m}}{\text{IBW at }0\text{ dS}/\text{m}}\;\times \;100$$

Regression analysis was performed to determine the extent of the relationship between IBW and salinity levels at different phases. Stepwise regression was also performed to find out the critical phase for salinity stress to IBW. Regression analysis and visualization were performed by using the “ggplot2” package (Wickham, 2009) in the “R” platform (R Core Team R, 2021).

## 3 Results

### 3.1 First-year observation

#### 3.1.1 Germination and subsequent growth repression percentage

Soil salinity markedly influenced the germination percentage of onion (**Table 1**). Germination percentage was recorded the highest in Ac Gaz 379 (98%) followed by Ac Bog 418 (90%), Ac Bog 432 (84%), and Ac Bog 422 (78%) compared to the rest of the germplasm, whereas the lowest germination (36%) was recorded in BARI Piaz-4 (check cultivar) under 8 dS m^−1^. Under normal water treatment, germination percentage was found to be higher in Ac Gaz 379 (100%), Ac Bog 428 (92%), Ac Bog 423 (92%), Ac Bog 422 (90%), Ac Bog 421 (94%), Ac Bog 420 (90%), Ac Bog 422 (96%), and Ac Bog 409 (90%) than the rest of the germplasm, and the lowest germination (60%) was recorded in Ac Bog 417. Under 10 dS m^−1^, germination percentage ranged from 36% (Ac Bog 418) to 92% (Ac Gaz 379). On the other hand, the highest germination percentage was recorded in Ac Gaz 379 (100%), which was followed by Ac Bog 418 (76%), Ac Bog 423 (72%), and Ac Bog 424 (72%), and the lowest germination was recorded in BARI Piaz-4, which was only 14% under the 12 dS m^−1^ salinity level. Finally, after subjecting all levels of salinity (0, 8, 10, and 12 dS m^−1^), germination percentage was found to range from 38% to 97.50%.

**Table 1:** Scanning the germination ability of onion genotypes at different salinity stress levels in the first year.

Genotype	Germination percentage (%)					Growth repressed (%)				
	C (0 dS m^−1^)	8 dS m^−1^	10 dS m^−1^	12 dS m^−1^	Mean	C (0 dS m^−1^)	8 dS m^−1^	10 dS m^−1^	12 dS m^−1^	Mean
Ac Bog 409	90	66	62	44	65.5	6	14	24	42	21.5
Ac Bog 410	74	64	64	56	64.5	8	8	20	18	13.5
Ac Bog 411	78	62	44	50	58.5	8	6	26	24	16
Ac Bog 412	86	50	66	26	57	8	12	24	22	16.5
Ac Bog 414	86	66	48	56	64	4	16	16	24	15
Ac Bog 415	64	68	48	48	57	6	16	22	18	15.5
Ac Bog 416	84	74	76	36	67.5	6	6	26	44	20.5
Ac Bog 417	60	68	80	66	68.5	8	14	26	36	21
Ac Bog 418	96	90	36	76	74.5	4	4	6	4	4.5
Ac Bog 419	86	58	70	52	66.5	8	16	14	20	14.5
Ac Bog 420	90	74	52	56	68	4	8	6	12	7.5
Ac Bog 421	94	72	68	50	71	4	8	6	24	10.5
Ac Bog 422	90	78	48	54	67.5	4	18	10	22	13.5
Ac Bog 423	92	72	72	72	77	2	8	22	12	11
Ac Bog 424	84	42	52	72	62.5	6	12	28	12	14.5
Ac Bog 425	82	74	64	74	73.5	4	6	10	16	9
Ac Bog 426	82	66	54	52	63.5	12	8	12	26	14.5
Ac Bog 427	74	60	60	46	60	8	18	12	22	15
Ac Bog 428	92	74	46	46	64.5	8	12	30	12	15.5
Ac Bog 429	82	58	72	44	64	6	12	40	14	18
Ac Bog 430	86	72	74	54	71.5	4	16	16	16	13
Ac Bog 431	80	70	44	70	66	6	4	14	16	10
Ac Bog 432	84	82	50	56	68	2	4	10	18	8.5
Ac Gaz 379	100	98	92	100	97.5	2	0	8	2	3
BARI Piaz-4	66	36	36	14	38	8	16	40	26	22.5
Mean	83.28	67.76	59.12	54.8	66.24	5.84	10.48	18.72	20.08	13.78
SD	9.8	13.28	14.34	17.28		2.44	5.1	9.86	9.96	
Min	60	36	36	14		2	0	6	2	
Max	100	98	92	100		12	18	40	44	

SD, standard deviation; Min, minimum; Max, maximum; C, control treatment, 0 dS m^−1^.

Under all levels of salinity (8, 10, and 12 dS m^−1^), the growth of germinated onion bulb was repressed in a significant way (**Table 1**) compared to untreated control as it ranged from 1% to 6% and 1% to 22% in control and 12 dS m^−1^ treatment, respectively. After imposing 8 dS m^−1^ salinity, the lowest growth repression % was recorded in Ac Gaz 379 (0) and the highest was recorded in Ac Bog 422 (9). After exerting 10 dS m^−1^ salinity, the lowermost growth repression % was noted in Ac Gaz 418 (3), Ac Gaz 420 (3), and Ac Gaz 421 (3), and the highest was noted in Ac Bog 429 (20) and BARI Piaz-4 (20). When 12 dS m^−1^ salinity was applied, the lowest growth repression % was found in Ac Gaz 379 (1) and the highest was found in Ac Bog 416 (22). Considering the mean values of all treatments (0, 8, 10, and 12 dS m^−1^), the lowest growth repression % was observed in Ac Gaz 379 (1.5) and the highest was observed in BARI Piaz-4 (11.25).

**Figure 1:**
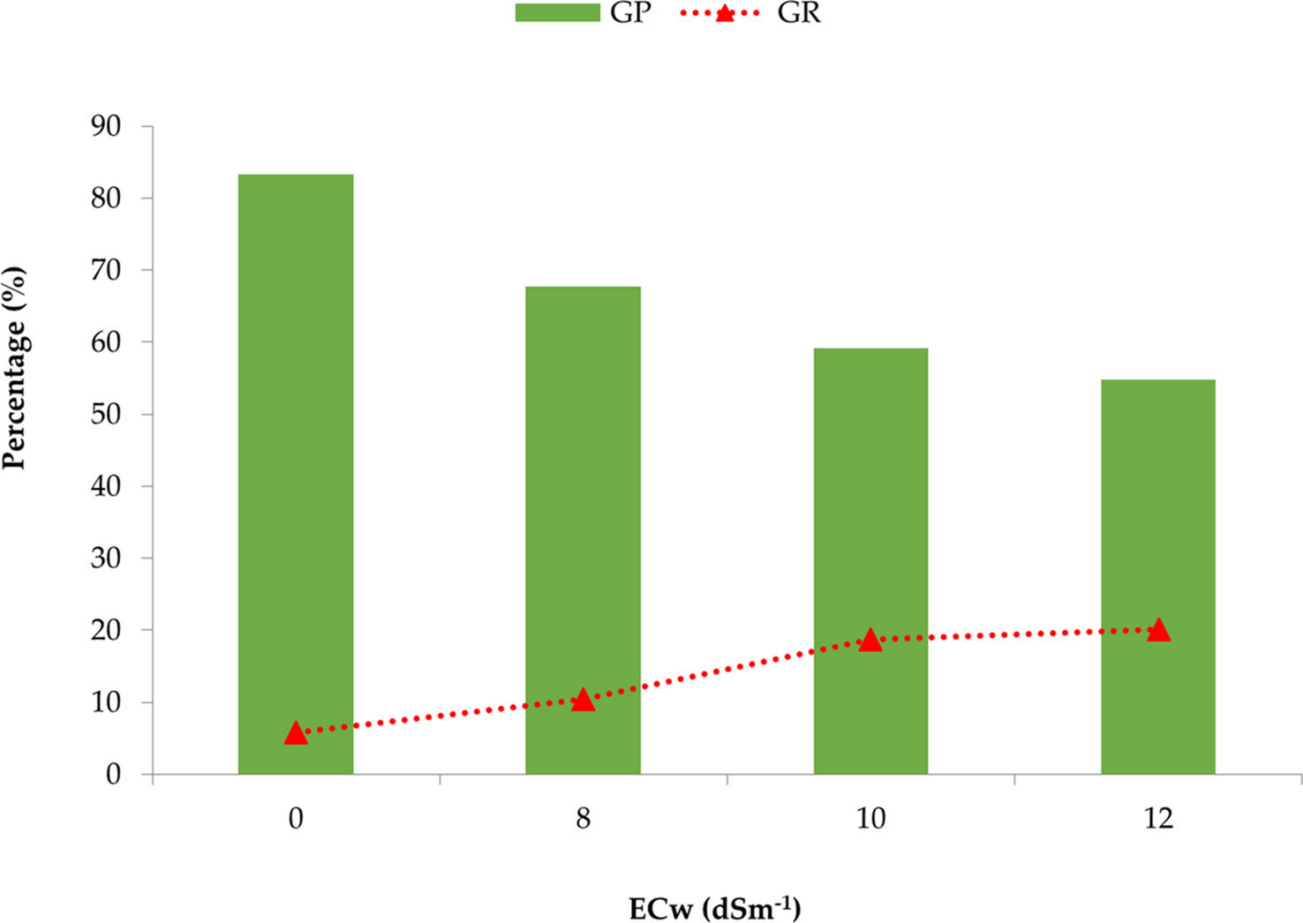
Comparison of the mean germination percentage (GP) and growth repression (GR) of 25 onion genotypes at different concentrations or salinity levels of irrigation water.

From **Figure 1**, it was revealed that the germination percentage gradually decreased with the increase of irrigation water salinity concentration. In contrast, the subsequent growth repression among the genotypes was minimum in the control treatment (0 dS m^−1^), and it was increased along the salinity level. Ultimately, the highest percentage of seized growth or death of germinated seed was observed at the 12 dS m^−1^ treatment.

### 3.2 Second-year observation

#### 3.2.1 Analysis of variance

Variance analysis was carried out among the studied traits of the second-year study and substantial variations (*p*< 0.01) were observed (**Table S2**). Variance due to genotypes (σ^2^g) and salinity levels (σ^2^s) were significant for all the studied traits, while variance due to genotypes:salinity level (σ^2^g×s) was significant for all the traits except MLL.

#### 3.2.2 Progression of soil salinity

**Figure 2:**
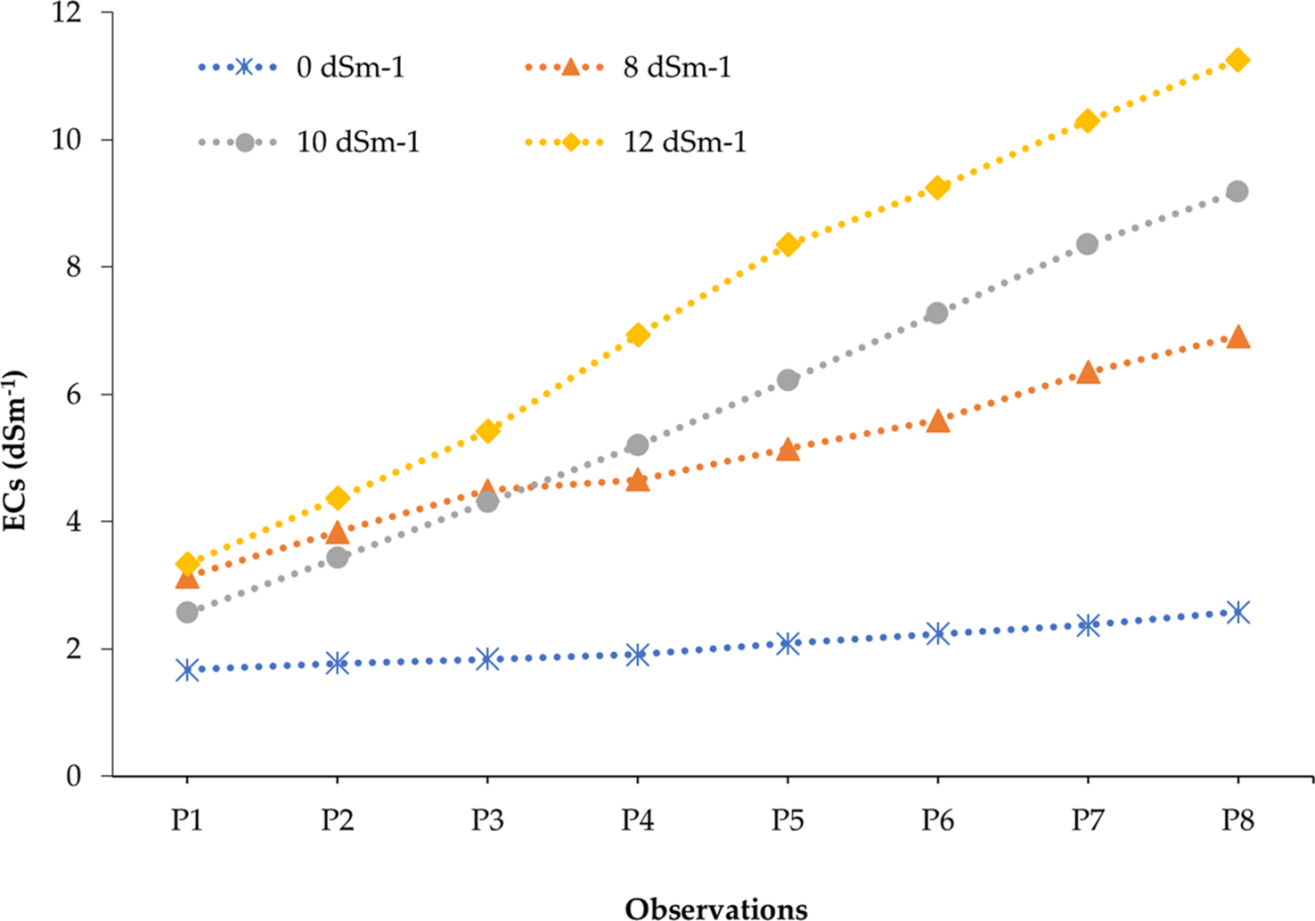
Development of soil salinity (EC_s_) over the periods of the onion life cycle at different salinity concentrations. All the data points are the mean values of EC_s_ recorded in replicated experimental pots representing 25 different onion genotypes (150 pots).

**Figure 3:**
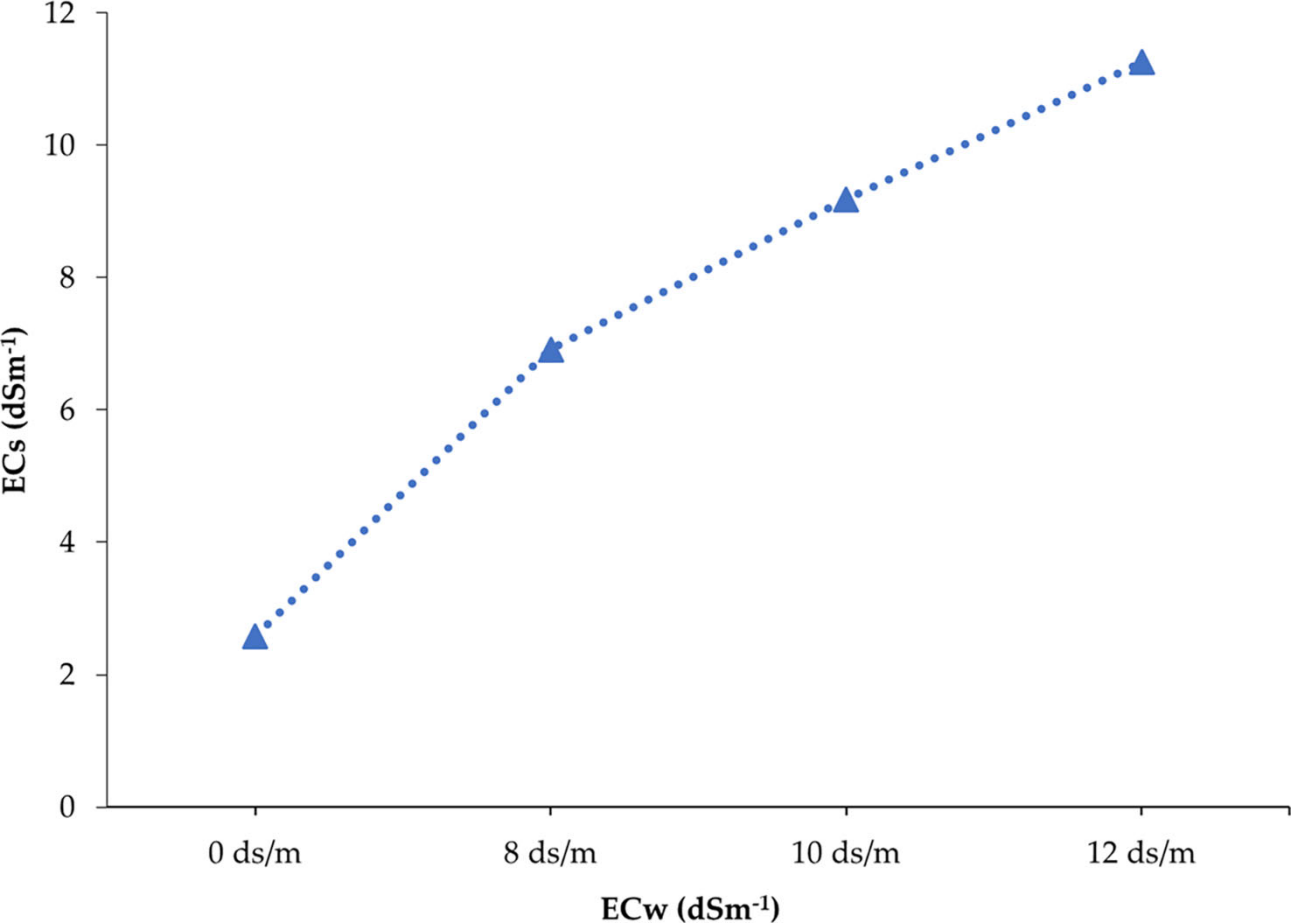
Soil salinity development after the application of different treatments at the end of the cropping season. Each point represents the mean of EC_s_ values recorded in replicated experimental pots of 25 genotypes (150 pots).

The studied soil was non-saline during the entire growing period of onion as the salinity range of soil under untreated control ranged from 1.67 to 2.58 dS m^−1^ (**Table S4**). In contrast, soil salinity was gradually built up with the advancement of different phases of onion plants depending on the concentration of salt in the solution (0, 8, 10, and 12 dS m^−1^) after eight spells of application (**Tables S5**-**S7**). After the application of irrigation water having 8, 10, and 12 dS m^−1^ of salinity, it was found that minimum levels of ECs (electrical conductivity in pot soil) were recorded in the first phase and maximum levels were recorded in the eighth phase. For example, 3.14, 2.57, and 3.33 dS m^−1^ were recorded from the first phase of 8, 10, and 12 dS m^−1^ treatments, respectively, whereas 6.92, 9.18, and 11.26 dS m^−1^ were obtained from 8, 10, and 12 dS m^−1^ treatments at the eighth phase, respectively. In the last phase of crops, soil salinity was increased by 168.22%, 255.81%, and 336.43% in 8, 10, and 12 dS m^−1^ treatments compared to the control treatment (0 dS m^−1^), respectively. The salinity profile in pot soil was in ascending order from the start to the end of the crop cycle, i.e., first to eighth phase (**Figure 2**). The soil salinity levels (EC_s_) reached at the eighth phase in all treatments (8, 10, and 12 dS m^−1^) were lower than those of the respective irrigation water salinity (**Figure 3**). However, it was expected to be the same at the irrigation treatments in an equilibrium state.

#### 3.2.3 Impact of different levels of salinity on leaf-associated traits

Drying out of onion plant leaves was markedly influenced by the different levels (0, 8, 10, and 12 dS m^−1^) of salinity (**Table 2** and **Table S3**). Almost 100% of the seedlings of onion plants were green under non-saline treatment, but at 8 dS m^−1^, no plants with dried leaves were found in Ac Bog 414, Ac Bog 415, Ac Bog 416, Ac Bog 417, Ac Bog 418, Ac Bog 419, Ac Bog 420, Ac Bog 422, Ac Bog 424, and Ac Bog 425 where the highest percentage of plants with dried leaves (PPDL) was recorded in Ac Bog 410 (100%), Ac Bog 432 (100%), and Ac Bog 428 (83.33%). After applying 10 dS m^−1^ salinity, no plants with dried leaves were found in Ac Bog 418, Ac Bog 420, and Ac Bog 424, but the highest PPDL was found in Ac Bog 432 (100%) and Ac Gaz 379 (83.33%).

**Table 2:** Performance of onion genotypes for greenness-related traits under different salinity stress levels in pot culture in the second year.

Genotype	Plants with dried leaves (%)					Green plants (%)				
	C (0 dS m^−1^)	8 dS m^−1^	10 dS m^−1^	12 dS m^−1^	Mean	C (0 dS m^−1^)	8 dS m^−1^	10 dS m^−1^	12 dS m^−1^	Mean
Ac Bog 409	0 (1.65)^*^	33.33 (35.26)	33.33 (35.26)	33.33 (35.26)	25 (26.86)	100 (88.35)	66.67 (54.74)	83.33 (71.54)	66.67 (54.74)	79.17 (67.34)
Ac Bog 410	0 (1.65)	100 (88.35)	66.67 (54.74)	100 (88.35)	66.67 (58.27)	100 (88.35)	0 (1.65)	50 (45)	0 (1.65)	37.5 (34.16)
Ac Bog 411	0 (1.65)	50 (45)	66.67 (54.74)	100 (88.35)	54.17 (47.43)	100 (88.35)	33.33 (35.26)	33.33 (35.26)	0 (1.65)	41.67 (40.13)
Ac Bog 412	0 (1.65)	33.33 (35.26)	16.67 (18.46)	0 (1.65)	12.5 (14.26)	100 (88.35)	50 (45)	83.33 (71.54)	66.67 (54.74)	75 (64.91)
Ac Bog 414	0 (1.65)	0 (1.65)	16.67 (18.46)	33.33 (35.26)	12.5 (14.26)	100 (88.35)	100 (88.35)	83.33 (71.54)	66.67 (54.74)	87.5 (75.74)
Ac Bog 415	0 (1.65)	0 (1.65)	16.67 (18.46)	50 (45)	16.67 (16.69)	100 (88.35)	100 (88.35)	83.33 (71.54)	33.33 (35.26)	79.17 (70.87)
Ac Bog 416	0 (1.65)	0 (1.65)	16.67 (18.46)	83.33 (71.54)	25 (23.33)	100 (88.35)	100 (88.35)	66.67 (54.74)	16.67 (18.46)	70.83 (62.47)
Ac Bog 417	0 (1.65)	0 (1.65)	16.67 (18.46)	66.67 (54.74)	20.83 (19.13)	100 (88.35)	100 (88.35)	83.33 (71.54)	33.33 (35.26)	79.17 (70.87)
Ac Bog 418	0 (1.65)	0 (1.65)	0 (1.65)	33.33 (35.26)	8.33 (10.06)	100 (88.35)	50 (45)	100 (88.35)	66.67 (54.74)	79.17 (69.11)
Ac Bog 419	0 (1.65)	0 (1.65)	16.67 (18.46)	16.67 (18.46)	8.33 (10.06)	100 (88.35)	83.33 (71.54)	83.33 (71.54)	83.33 (71.54)	87.5 (75.74)
Ac Bog 420	0 (1.65)	0 (1.65)	0 (1.65)	100 (88.35)	25 (23.33)	100 (88.35)	100 (88.35)	100 (88.35)	16.67 (18.46)	79.17 (70.87)
Ac Bog 421	0 (1.65)	33.33 (35.26)	16.67 (18.46)	16.67 (18.46)	16.67 (18.46)	100 (88.35)	66.67 (54.74)	83.33 (71.54)	66.67 (54.74)	79.17 (67.34)
Ac Bog 422	0 (1.65)	0 (1.65)	16.67 (18.46)	0 (1.65)	4.17 (5.86)	100 (88.35)	83.33 (71.54)	66.67 (54.74)	100 (88.35)	87.5 (75.74)
Ac Bog 423	0 (1.65)	16.67 (18.46)	33.33 (35.26)	50 (45)	25 (25.09)	100 (88.35)	83.33 (71.54)	66.67 (54.74)	50 (45)	75 (64.91)
Ac Bog 424	0 (1.65)	0 (1.65)	0 (1.65)	33.33 (35.26)	8.33 (10.06)	100 (88.35)	100 (88.35)	100 (88.35)	66.67 (54.74)	91.67 (79.94)
Ac Bog 425	0 (1.65)	0 (1.65)	16.67 (18.46)	16.67 (18.46)	8.33 (10.06)	100 (88.35)	83.33 (71.54)	66.67 (54.74)	83.33 (71.54)	83.33 (71.54)
Ac Bog 426	0 (1.65)	16.67 (18.46)	33.33 (35.26)	33.33 (35.26)	20.83 (22.66)	100 (88.35)	66.67 (54.74)	66.67 (54.74)	33.33 (35.26)	66.67 (58.27)
Ac Bog 427	16.67 (18.46)	50 (45)	66.67 (61.8)	83.33 (71.54)	54.17 (49.2)	83.33 (71.54)	33.33 (28.19)	33.33 (35.26)	16.67 (18.46)	41.67 (38.36)
Ac Bog 428	16.67 (18.46)	83.33 (71.54)	66.67 (54.74)	33.33 (35.26)	50 (45)	83.33 (71.54)	16.67 (18.46)	33.33 (35.26)	66.67 (54.74)	50 (45)
Ac Bog 429	0 (1.65)	16.67 (18.46)	16.67 (18.46)	16.67 (18.46)	12.5 (14.26)	100 (88.35)	66.67 (54.74)	66.67 (54.74)	66.67 (54.74)	75 (63.14)
Ac Bog 430	0 (1.65)	16.67 (18.46)	33.33 (35.26)	50 (45)	25 (25.09)	100 (88.35)	66.67 (61.81)	50 (45)	0 (1.65)	54.17 (49.2)
Ac Bog 431	0 (1.65)	66.67 (54.74)	50 (45)	16.67 (18.46)	33.33 (29.96)	100 (88.35)	16.67 (18.46)	33.33 (35.26)	66.67 (54.74)	54.17 (49.2)
Ac Bog 432	0 (1.65)	100 (88.35)	100 (88.35)	100 (88.35)	75 (66.67)	100 (88.35)	0 (1.65)	0 (1.65)	0 (1.65)	25 (23.33)
Ac Gaz 379	0 (1.65)	50 (45)	83.33 (71.54)	66.67 (54.74)	50 (43.23)	100 (88.35)	50 (45)	16.67 (18.46)	33.33 (35.26)	50 (46.77)
BARI Piaz-4	0 (1.65)	66.67 (54.74)	16.67 (18.46)	0 (1.65)	20.83 (19.13)	100 (88.35)	33.33 (35.26)	83.33 (71.54)	16.67 (18.46)	58.33 (53.4)
Mean	1.33 (3)	29.33 (27.55)	32.67 (31.2)	45.33 (41.99)	27.17 (25.94)	98.67 (87)	62 (54.84)	64.67 (56.68)	44.67 (39.62)	67.5 (59.53)
CV %	50.83				–	21.88				–
LSD (0.05)	5.23				–	12.93				–

CV, coefficient of variation; LSD (0.05), least significant difference; C, control treatment, 0 dS m^−1^; ^*^data in parentheses represent the transformed value of the corresponding data.

At 12 dS m^−1^ salinity, no plants with dried leaves were found in Ac Bog 412, Ac Bog 422, and BARI Piaz-4, but the highest PPDL was found in Ac Bog 411 (100%), Ac Bog 412 (100%), Ac Bog 420 (100%), and Ac Bog 432 (100%). Mean PPDL ranged from 4.17% (Ac Bog 422) to 75% (Ac Bog 432) after subjecting all levels (0, 8, 10, and 12 dS m^−1^) of salinity.

The percentage of green plants (PGP) was noticeably influenced by imposing salinity (**Table 2** and **Table S3**). Almost all studied plants remain green under control treatments, but under 8 dS m^−1^, 100% of plants were dead in Ac Bog 410 and Ac Bog 432 genotypes where, under the same treatment, the highest PPG (100%) was observed in Ac Bog 414, Ac Bog 415, Ac Bog 416, Ac Bog 420, and Ac Bog 424. Under 10 dS m^−1^ of salinity, the highest percentage (100%) of green plants was found in Ac Bog 418, Ac Bog 420, and Ac Bog 424 where the lowest number of green plants was recorded in Ac Bog 432 (0%). Under 12 dS m^−1^ of salinity, in the Ac Bog 422 genotype, the highest percentage (100%) of green plants was noted and the lowest (0%) was obtained from Ac Bog 410, Ac Bog 411, Ac Bog 430, and Ac Bog 432. After subjecting all levels (0, 8, 10, and 12 dS m^−1^) of salinity, PGP was found to range from 1.5 to 5.25.

MLL was prominently affected by imposing salinity (**Table 3** and **Table S3**). The MLL (cm) of onion seedlings ranged from 13.67 to 26.00 under untreated control treatment. The highest MLL (cm) under 8 dS m^−1^ was recorded in Ac Bog 409 (25.67), which was followed by Ac Bog 415 (24.33), Ac Bog 414 (23.80), and Ac Bog 416 (23.60), and the lowest MLL was found in Ac Bog 432 (0.0001). Under 10 dS m^−1^, MLL (cm) was noted to be the highest in Ac Bog 422 (21.17), which was followed by Ac Bog 416 (20.67) and Ac Bog 423 (20.30), and the lowest was obtained in Ac Bog 410 (7.50). The highest MLL (cm) under 12 dS m^−1^ salinity was recorded in Ac Bog 414 (23.40) and the lowest was recorded in Ac Bog 432 (0.0001). After subjecting all levels of treatments (0, 8, 10, and 12 dS m^−1^), the average MLL of onion seedlings was between 9.44 and 22.76 cm.

**Table 3:** Performance of onion genotypes for leaf-related traits under different salinity stress levels in pot culture in the second year.

Genotypes	Maximum leaf length					Number of leaves				
	C (0 dS m^−1^)	8 dS m^−1^	10 dS m^−1^	12 dS m^−1^	Mean	C (0 dS m^−1^)	8 dS m^−1^	10 dS m^−1^	12 dS m^−1^	Mean
Ac Bog 409	25.67	25.25	16.17	17.6	21.17	6.17	4.00	5.17	4.17	4.88
Ac Bog 410	23.5	11.33	7.5	10	13.08	2.83	4.17	1.33	2.83	2.79
Ac Bog 411	22	19.5	11.5	0.0001	13.25	2.83	4.00	2.83	1.83	2.87
Ac Bog 412	25	17	15.8	17.2	18.75	5.33	5.33	4.00	4.50	4.79
Ac Bog 413	21.5	18.83	15.67	8.4	16.10	6.00	4.17	4.83	4.50	4.29
Ac Bog 414	24	23.8	19.83	23.4	22.76	5.17	5.67	5.83	4.67	5.54
Ac Bog 415	24.5	24.33	19	19.4	21.81	4.67	5.00	6.00	4.67	5.21
Ac Bog 416	25.67	23.6	20.67	12	20.49	6.00	4.50	6.67	3.50	4.83
Ac Bog 417	23.33	22.33	17.5	21.8	21.24	3.67	5.50	5.50	4.83	5.46
Ac Bog 419	24.33	14.5	20	18.17	19.25	6.33	4.83	3.17	5.00	4.83
Ac Bog 420	25.83	17.5	18.17	17.5	19.75	6.17	5.83	3.83	4.67	5.13
Ac Bog 421	18.8	18.2	14.83	14.33	16.54	4.50	5.17	5.50	4.00	4.79
Ac Bog 422	24.33	22	21.17	15.33	20.71	5.83	5.17	4.83	4.50	5.08
Ac Bog 423	22.17	20.6	20.3	20.25	20.83	7.17	5.00	5.83	4.83	5.71
Ac Bog 424	25.83	21.67	18.5	20.75	21.69	6.17	5.67	3.17	5.50	5.13
Ac Bog 425	20.17	19.67	19.37	18.5	19.43	6.33	5.83	3.33	4.33	4.96
Ac Bog 426	24.2	22.8	17.83	12.67	19.38	6.00	5.00	5.00	4.00	5.00
Ac Bog 427	19.67	19	12.33	12	15.75	5.33	3.17	5.67	3.17	4.33
Ac Bog 428	13.67	12.5	10.67	8.25	11.27	1.67	2.50	3.00	3.33	2.63
Ac Bog 429	26	23.33	13.83	16.4	19.89	4.83	5.50	4.67	4.83	4.96
Ac Bog 430	21.17	17	19.8	17.4	18.84	5.67	4.83	2.67	4.83	4.50
Ac Bog 431	21.75	20	18.5	15	18.81	2.50	3.00	4.67	3.83	3.50
Ac Bog 432	22.75	0.0001	15	0.0001	9.44	3.17	2.83	2.00	2.67	2.67
Ac Gaz 379	20.33	19	15.67	10	16.25	3.00	2.83	5.33	2.83	3.50
BARI Piaz-4	21.5	18	10	15.25	16.19	2.50	5.00	2.67	4.83	3.75
Mean	22.69	17.17	20.72	15.51	19.11	4.8	4.3	4.58	4.11	4.45
CV%	20.76				–	20.97				–
LSD (0.05)	–				–	0.92				

CV, coefficient of variation; LSD (0.05), least significant different; C, control treatment; 0 dS m^−1^.

The number of leaves (NL) was markedly influenced by the application of salinity (0, 8, 10, and 12 dS m^−1^) treatments (**Tables 3** and **S3**). The number of leaves of onion seedlings ranged from 1.67 to 7.17 under untreated control treatment. Under the 8 dS m^−1^ salinity level, the highest NL was recorded in Ac Bog 420 (5.83) and Ac Bog 425 (5.83), and the lowest NL was recorded in Ac Bog 428 (2.50). The highest NL was found in Ac Bog 416 (6.67) under 10 dS m^−1^ salinity, whereas the lowest NL was found in Ac Bog 410 (1.33). Under 12 dS m^−1^ salinity treatment, the lowest NL was observed in Ac Bog 411 (1.83), whereas the highest NL was found in Ac Bog 424 (5.50). Considering the mean values of NL over the different salinity levels, the leaf number varied between 2.63 and 5.71.

#### 3.2.4 Impact of different levels of salinity on bulb-associated traits

The BL (cm) of onion seedlings was decisively influenced by the salinity treatments (**Table 4** and **S3**).

**Table 4:** Performance of onion genotypes for bulb-related traits under different salinity stress levels in pot culture in the second year.

Genotype	Bulb length (mm)					Bulb diameter (mm)					Individual bulb weight (g)				
	C (0 dS m^−1^)	8 dS m^−1^	10 dS m^−1^	12 dS m^−1^	Mean	C (0 dS m^−1^)	8 dS m^−1^	10 dS m^−1^	12 dS m^−1^	Mean	C (0 dS m^−1^)	8 dS m^−1^	10 dS m^−1^	12 dS m^−1^	Mean
Ac Bog 409	18.8	35.92	34.56	48.78	34.52	39.56	15.7	24.26	22.2	25.44	22.4	22.2	8.75	8.85	15.55
Ac Bog 410	19.46	15.54	17.64	30.92	20.88	21	19.02	19.48	18.42	19.48	14.2	5.25	4.75	5.25	7.35
Ac Bog 411	15.2	15.98	26.34	37.18	23.68	21.14	12.14	20.74	13.98	17	14.25	7.45	2.8	2.6	6.8
Ac Bog 412	15.38	29.76	31.48	30.3	26.72	16	11.9	16.56	13.96	14.6	12.75	4.9	5.8	6.35	7.45
Ac Bog 413	20.8	18.16	26.04	44.28	27.32	24.72	18.84	9.56	9.34	15.62	25.95	9.4	1.65	1.65	9.65
Ac Bog 414	27.76	26.92	44.02	43.02	35.44	27.8	23.36	22.82	22.64	24.16	30.5	12	11.4	11.85	16.4
Ac Bog 415	28.36	37.08	37.72	40.74	35.98	23.74	26.1	24.3	19.02	23.28	25.55	11.4	6.7	4.1	11.95
Ac Bog 416	25.66	23.84	37.96	44.76	33.06	24.56	20.52	26.5	14.22	21.44	27.2	16.65	3.4	3.65	12.7
Ac Bog 417	28.04	50.2	47.38	47.66	43.32	30.3	22.3	27.54	24.96	26.28	22.85	19.7	5.8	3.6	13
Ac Bog 419	36.08	36.44	36.76	30.18	34.86	14.38	35.72	17.4	16.86	21.1	39.05	5.35	7.45	7.25	14.75
Ac Bog 420	25.32	45.26	44.62	31.66	36.72	20.12	23.62	31.46	29.4	26.14	25.85	6.9	16.2	17	16.5
Ac Bog 421	17.5	27	29.24	35.32	27.26	17.54	15.4	19.68	20.92	18.38	18.45	5.1	5.75	5.4	8.65
Ac Bog 422	27.14	34.46	37.12	33.02	32.94	24.3	27.84	23.56	24.3	25	30.85	10.7	8	8.55	14.5
Ac Bog 423	38.74	34.08	35.66	33.72	35.56	17.06	34	15.34	14.16	20.14	38.15	5.5	4	2.9	12.65
Ac Bog 424	27.9	49.58	47.2	46.46	42.78	20.64	21.62	27.2	25.7	23.78	28.35	2.1	15.6	14.65	15.15
Ac Bog 425	29.72	38.86	40.22	36.28	36.26	27.7	30.08	23.1	23.98	26.22	33.35	12	7.25	7.05	14.9
Ac Bog 426	36.7	33.16	34.76	54.46	39.78	33.26	38.36	14.46	13.26	24.84	35.2	3.55	3.85	3.85	11.6
Ac Bog 427	22.94	30.62	24	27.66	26.3	26.56	16.9	18.28	11.44	18.3	18.2	17.7	2.75	3.15	10.45
Ac Bog 428	16.14	23.2	22.64	23.64	21.4	11.2	15.02	20.84	8.32	13.84	25.55	9	1.5	1.4	9.35
Ac Bog 429	17.64	27	30.82	43.72	29.8	26.62	14.52	31.68	15.38	22.04	20.3	11.75	4.15	4.7	10.25
Ac Bog 430	24.88	39.74	37.76	34.64	34.26	25.9	24.84	24.84	25.02	25.14	30.5	17.7	8.05	8.7	16.25
Ac Bog 431	19.54	24.56	33.04	39.18	29.08	20.78	17.18	16.02	17.48	17.86	17.65	9.25	3.9	3.35	8.55
Ac Bog 432	18.18	6.84	8.52	17	12.64	18.26	15.62	7.02	7.52	12.1	7.65	3.7	2.2	2.1	3.9
Ac Gaz 379	17.22	27.04	30.26	26.36	25.22	16.34	15.9	29.26	18.44	19.98	23.7	3.1	4.55	4.45	8.95
BARI Piaz-4	12.76	36.04	29.82	28.46	26.78	16.44	10.4	14.28	14.36	13.88	9.05	3.5	3.55	4.15	5.05
Mean	23.51	30.69	33.02	36.38	30.90	22.64	21.08	21.05	17.81	20.64	23.90	9.43	5.99	5.86	11.29
CV%	20.52					35.11					48.23				
LSD (0.05)	3.11					3.55					1.07				

CV, coefficient of variation; LSD (0.05), least significant different; C, control treatment; 0 dS m^−1^.

BL (cm) ranged from 12.76 to 38.74 in the control treatment. The highest BL (cm) was found in Ac Bog 417 (50.2), which was followed by Ac Bog 424 (49.58), and the lowest was recorded in Ac Bog 432 (6.84) under 8 dS m^−1^ salinity. Moreover, under 10 dS m^−1^ salinity, the highest BL (cm) was found in Ac Bog 417 (47.38), which was followed by Ac Bog 424 (47.2), Ac Bog 420 (44.62), and Ac Bog 414 (44.02), and the lowest was recorded in Ac Bog 432 (8.52).

On the other hand, the highest BL (cm) was obtained from Ac Bog 426 (54.46), and the lowest was recorded in Ac Bog 432 (17) under 12 dS m^−1^ salinity. After assessing the mean of BL (cm) of all treatments, BL ranged from 12.64 to 43.32 cm.

The BD (cm) of onion seedlings was remarkably influenced by the salinity treatments (**Tables 4** and **S3**). BD (cm) ranged from 11.2 to 39.56 in the untreated control. The highest BD (cm) under 8 dS m^−1^ salinity was observed in Ac Bog 426 (38.36) and the lowest was recorded in BARI Piaz-4 (10.4). Furthermore, under 10 dS m^−1^ salinity, the highest BD (cm) was found in Ac Bog 429 (31.68), which was followed by Ac Bog 420 (31.46), and the lowest was recorded in Ac Bog 432 (7.02). In contrast, the highest BD (cm) under 12 dS m^−1^ salinity was found in Ac Bog 420 (29.4) and the lowest was recorded in Ac Bog 432 (7.52). After evaluating the mean of BD (cm) of all treatments, the average BD ranged from 12.1 to 26.28 cm.

The IBW (g) of onion seedlings was markedly influenced by the salinity treatments (**Tables 4** and **S3**). IBW (g) oscillated from 7.65 to 38.15 in the untreated control. The highest IBW (g) under 8 dS m^−1^ salinity was observed in Ac Bog 409 (22.2) and the lowest was recorded in Ac Bog 424 (2.1). Moreover, under 10 dS m^−1^ salinity, the highest IBW (g) was found in Ac Bog 420 (16.2), which was followed by Ac Bog 424 (15.6), and the lowest was noted in Ac Bog 428 (1.5). In comparison, the highest IBW (g) under 12 dS m^−1^ salinity was found in Ac Bog 420 (17) and the lowest was documented in Ac Bog 428 (1.4). The mean IBW (g) of all salinity treatments varied between 3.9 and 16.5 g for all the studied genotypes.

#### 3.2.5 Associated indices of soil salinity stress

ShTI, STI, and PYR were distinctly influenced by the application of different levels of salinity in comparison with the untreated control (**Tables 5** and **6**). The ShTI under 8 dS m^−1^ ranged from 0.00 to 99.31 with a mean of 88.62 (**Table 5**). The highest ShTI was recorded in Ac Bog 415, which was followed by Ac Bog 414 (99.17) and Ac Bog 409 (98.36), and the lowest ShTI was recorded in Ac Bog 432 under 8 dS m^−1^ salinity treatment. Under 10 dS m^−1^, the ShTI ranged from 31.91 to 96.03 with a mean of 75.56. The highest ShTI was documented in Ac Bog 425, and the lowest ShTI was documented in Ac Bog 410. The mean ShTI under 12 dS m^−1^ was 63.56 with a range from 0.00 to 97.50, where the highest ShTI was recognized in Ac Bog 414 but the lowest was recognized in Ac Bog 411 and Ac Bog 432.

**Table 5:** Stress indices and their ranks for the different studied onion genotypes under different salinity treatments in pot culture in the second year.

Genotype	ShTI_8 dS m^−1^	R	ShTI_10 dS m^−1^	R	ShTI_12 dS m^−1^	R	MR	STI_8 dS m^−1^	R	STI_10 dS m^−1^	R	STI_12 dS m^−1^	R	MR
Ac Bog 409	98.36	3	62.99	20	68.56	13	12	3.86	2	1.52	8	1.54	8	4.5
Ac Bog 410	48.21	24	31.91	25	42.55	22	23.67	0.58	20	0.52	19	0.58	18	14.25
Ac Bog 411	88.64	16	52.27	23	0	24	21	0.82	18	0.31	22	0.29	22	15.5
Ac Bog 412	68	21	63.2	19	68.8	12	17.33	0.48	22	0.57	17	0.63	17	14
Ac Bog 413	87.58	17	72.88	15	39.07	23	18.33	1.89	10	0.33	21	0.33	21	13
Ac Bog 414	99.17	2	82.63	6	97.5	1	3	2.84	6	2.7	3	2.81	3	3
Ac Bog 415	99.31	1	77.55	11	79.18	7	6.33	2.26	9	1.33	9	0.81	12	7.5
Ac Bog 416	91.94	12	80.52	8	46.75	21	13.67	3.52	3	0.72	15	0.77	14	8
Ac Bog 417	95.71	7	75.01	13	93.44	2	7.33	3.49	4	1.03	12	0.64	16	8
Ac Bog 419	59.6	23	82.2	7	74.68	9	13	1.62	14	2.26	4	2.2	4	5.5
Ac Bog 420	67.75	22	70.34	17	67.75	14	17.67	1.38	15	3.25	2	3.41	1	4.5
Ac Bog 421	96.81	5	78.88	9	76.22	8	7.33	0.73	19	0.82	14	0.77	13	11.5
Ac Bog 422	90.42	14	87.01	4	63.01	16	11.33	2.56	7	1.92	5	2.05	6	4.5
Ac Bog 423	92.92	10	91.57	3	91.34	4	5.67	1.63	13	1.18	10	0.86	10	8.25
Ac Bog 424	83.89	18	71.62	16	80.33	6	13.33	0.46	23	3.43	1	3.22	2	6.5
Ac Bog 425	97.52	4	96.03	1	91.72	3	2.67	3.11	5	1.88	7	1.83	7	4.75
Ac Bog 426	94.21	8	73.68	14	52.36	19	13.67	0.97	17	1.05	11	1.05	9	9.25
Ac Bog 427	96.59	6	62.68	21	61.01	17	14.67	2.5	8	0.39	20	0.45	20	12
Ac Bog 428	91.44	13	78.05	10	60.35	18	13.67	1.79	12	0.3	23	0.28	24	14.75
Ac Bog 429	89.73	15	53.19	22	63.08	15	17.33	1.85	11	0.65	16	0.74	15	10.5
Ac Bog 430	80.3	20	93.53	2	82.19	5	9	4.19	1	1.91	6	2.06	5	3
Ac Bog 431	91.95	11	85.06	5	68.97	11	9	1.27	16	0.53	18	0.46	19	13.25
Ac Bog 432	0	25	65.93	18	0	24	22.33	0.22	25	0.13	25	0.12	25	18.75
Ac Gaz 379	93.46	9	77.08	12	49.19	20	13.67	0.57	21	0.84	13	0.82	11	11.25
BARI Piaz-4	83.72	19	46.51	24	70.93	10	17.67	0.25	24	0.25	24	0.29	23	17.75
Mean	88.62		75.56		63.56			1.83		1.19		1.16		
SD	26.48		19.64		24.73			1.25		0.94		0.96		
Min	0.00		31.91		0.00			0.22		0.13		0.12		
Max	99.31		96.03		97.50			4.19		3.43		3.41		

ShTI, shoot tolerance index; STI, stress tolerance index; R, rank; MR, mean rank; SD, standard deviation; Min, minimum; Max, maximum.

**Table 6:** The yield reduction (%) and their ranks for the different studied onion genotypes under different saline water treatments.

Genotype	PYR_8 dS m^−1^	R	PYR_10 dS m^−1^	R	PYR_12 dS m^−1^	R	MR
Ac Bog 409	0.89	1	60.94	5	60.49	5	3.67
Ac Bog 410	63.03	14	66.55	7	63.03	7	9.33
Ac Bog 411	47.72	8	80.35	17	81.75	17	14.00
Ac Bog 412	61.57	13	54.51	3	50.2	3	6.33
Ac Bog 413	63.78	15	93.64	24	93.64	24	21.00
Ac Bog 414	60.66	11	62.62	6	61.15	6	7.67
Ac Bog 415	55.38	10	73.78	11	83.95	19	13.33
Ac Bog 416	38.79	4	87.5	21	86.58	21	15.33
Ac Bog 417	13.79	3	74.62	13	84.25	20	12.00
Ac Bog 419	86.3	22	80.92	19	81.43	16	19.00
Ac Bog 420	73.31	20	37.33	1	34.24	1	7.33
Ac Bog 421	72.36	19	68.83	8	70.73	8	11.67
Ac Bog 422	65.32	18	74.07	12	72.29	10	13.33
Ac Bog 423	85.58	21	89.52	23	92.4	23	22.33
Ac Bog 424	92.59	25	44.97	2	48.32	2	9.67
Ac Bog 425	64.02	16	78.26	15	78.86	13	14.67
Ac Bog 426	89.91	24	89.06	22	89.06	22	22.67
Ac Bog 427	2.75	2	84.89	20	82.69	18	13.33
Ac Bog 428	64.77	17	94.13	25	94.52	25	22.33
Ac Bog 429	42.12	6	79.56	16	76.85	12	11.33
Ac Bog 430	41.97	5	73.61	10	71.48	9	8.00
Ac Bog 431	47.59	7	77.9	14	81.02	14	11.67
Ac Bog 432	51.63	9	71.24	9	72.55	11	9.67
Ac Gaz 379	86.92	23	80.8	18	81.22	15	18.67
BARI Piaz-4	61.33	12	60.77	4	54.14	4	6.67
Mean	56.00		73.62		73.87		
SD	28.28		14.27		15.48		
Min	0.89		37.33		34.24		
Max	92.59		94.13		94.52		

PYR, percent yield reduction (over 0 dS m^−1^ treatment); R, rank; MR, mean rank; SD, standard deviation; Min, minimum; Max, maximum.

Under 8 dS m^−1^, the STI ranged from 0.22 to 4.19 with a mean of 1.79 (**Table 5**). Maximum STI was noted in Ac Bog 430, and the lowest was noted in Ac Bog 432 under 8 dS m^−1^ salinity treatment. The mean STI under 10 dS m^−1^ was 1.19 with a range of 0.13 to 3.43. The highest STI was documented in Ac Bog 424, which was followed by Ac Bog 420 (3.25), and the lowest was noted in Ac Bog 432. The STI under 12 dS m^−1^ ranged from 0.12 to 3.41 with a mean of 1.16. The highest STI under 12 dS m^−1^ salinity treatment was found in Ac Bog 420, which was followed by Ac Bog 424 (3.22), but the lowest was found in the Ac Bog 432 genotype. The mean PYR under 8 dS m^−1^ was 56.00 with a range of 0.89 to 92.59. The highest PYR under 8 dS m^−1^ salinity treatment was recorded in Ac Bog 424, which was followed by Ac Bog 426 (89.91) and Ac Bog 379 (86.92), and the lowest was noted in Ac Bog 409. The PYR under 10 dS m^−1^ ranged from 37.33 to 94.13 with a mean of 73.62. Under 10 dS m^−1^, the highest PYR was obtained in Ac Bog 428, which was followed by Ac Bog 413 (93.64) and Ac Bog 423 (89.52), and the lowest was obtained in Ac Bog 420. Under 12 dS m^−1^, the PYR ranged from 34.24 to 94.52 with a mean of 73.87. The highest PYR was found in Ac Bog 428, which was followed by Ac Bog 413 (93.64) and Ac Bog 423 (92.40), and the lowest was found in the Ac Bog 420 genotype.

#### 3.2.6 Ranking of the genotypes

Genotypes were ranked against salinity stress tolerance to select potential ones (**Table 5**). Based on different tolerance indices (ShTI and STI), genotypes were ranked, where dissimilarities in ranking positions were observed within a particular index for some of the potential genotypes. Thus, mean ranking (MR) was estimated for all the indices. Many of the genotypes are observed in the upper order among the MR of different indices, though differential results are also in place. Ranking and mean ranking were also estimated for yield reduction (%) (PYR), and a reflection of a similar ranking pattern from the previous result was observed (**Table 6**). Finally, to select better genotypes having good yield, better tolerance, and minimum yield loss under stress, ranking based on IBW and re-ranking of MR (of ShTI, STI, and PYR) was carried out (**Table 7**).

**Table 7:** Ranking of onion genotypes based on bulb weight, stress indices, and yield reduction (%) under different salinity treatments in pot culture in the second year.

Genotype	Rank based on				Occurrence* (in the top 10)
	IBW	MR (ShTI)	MR (STI)	MR (PYR)	
Ac Bog 409	4	8	2	1	4
Ac Bog 410	22	18	17	7	1
Ac Bog 411	23	16	19	13	0
Ac Bog 412	21	13	16	2	1
Ac Bog 413	16	15	14	18	0
Ac Bog 414	2	2	1	5	4
Ac Bog 415	12	4	6	12	2
Ac Bog 416	10	11	7	15	2
Ac Bog 417	9	5	7	11	3
Ac Bog 419	7	9	4	17	3
Ac Bog 420	1	14	2	4	3
Ac Bog 421	19	5	12	10	2
Ac Bog 422	8	7	2	12	3
Ac Bog 423	11	3	8	19	2
Ac Bog 424	5	10	5	8	4
Ac Bog 425	6	1	3	14	3
Ac Bog 426	13	11	9	20	1
Ac Bog 427	14	12	13	12	0
Ac Bog 428	17	11	18	19	0
Ac Bog 429	15	13	10	9	2
Ac Bog 430	3	6	1	6	4
Ac Bog 431	20	6	15	10	2
Ac Bog 432	25	17	21	8	1
Ac Gaz 379	18	11	11	16	0
BARI Piaz-4	24	14	20	3	1

IBW, individual bulb weight; MR, mean rank; ShTI, shoot tolerance index; STI, stress tolerance index; and PYR, percent yield reduction. *Total number of times a genotype is present within the top 10 at different rankings.

Genotypes’ position in those ranks was marked and their position within the top 10 was counted. The genotypes that present a maximum of four times in the top 10 were regarded as tolerant genotypes and those that present three times were denoted as moderate genotypes. The genotypes present four times in the top 10 were Ac Bog 409, Ac Bog 414, Ac Bog 424, and Ac Bog 430, whereas the genotypes that occurred three times within the top 10 were Ac Bog 417, Ac Bog 419, Ac Bog 420, Ac Bog 422, and Ac Bog 425. Those genotypes could be selected as salinity-tolerant candidates.

### 3.3 Regression study

**Figure 4:**
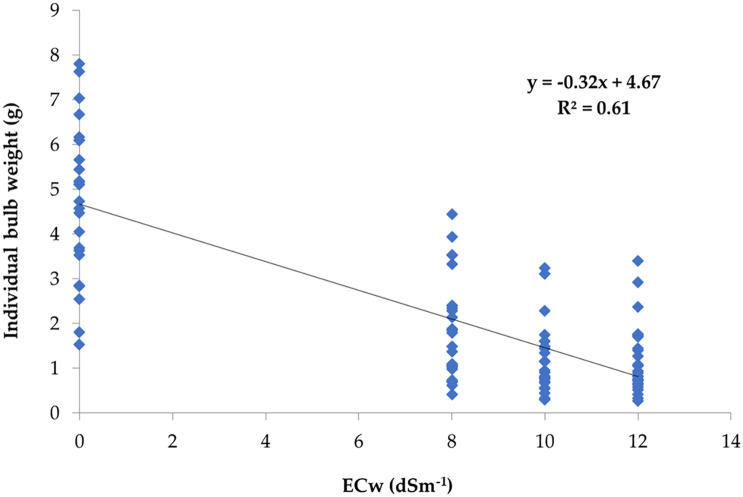
Regression of salinity concentration EC_w_ (dS m^−1^) of irrigation water on individual bulb weight (g).

A simple linear regression analysis was carried out involving salinity concentration (EC_w_) in irrigation water and IBW of onion corresponding to the salinity levels where IBW was considered as the dependent variable. The result showed that 61% of the IBW variation was accounted for by the irrigation water salinity levels (**Figure 4**).

The soil salinity levels (EC_s_) had significant effects on bulb formation and its subsequent development. The soil salinity levels at different phases were regressed on IBWs of the studied genotypes. The regression analysis helps to examine the strength of the relationship between dependent and independent variables. It helped to identify the relative importance of predictor variables in terms of contributing to the variation in dependent variables. The regression analysis showed that salinity levels at different phases negatively contributed to the IBW (**Table 8**).

**Table 8:** Regression analysis for IBW based on soil salinity developed at different phases.

Item Phase	SLR								MLR	SWR
	1	2	3	4	5	6	7	8		
b	−1.68	−1.36	−1.07	−0.76	−0.61	−0.54	−0.48	−0.44	–	–
R^2^	0.39	0.53	0.58	0.55	0.54	0.55	0.57	0.56	0.61	0.63
p-value	<0.001	<0.001	<0.001	<0.001	<0.001	<0.001	<0.001	<0.001	<0.001	<0.001

SLR = simple linear regression; MLR = multiple linear regression; SWR = stepwise regression; b = regression coefficient; R^2^ = coefficient of determination. Numbers 1 to 8 represent the different dates/phases when observation on EC_s_ was recorded.

**Figure 5:**
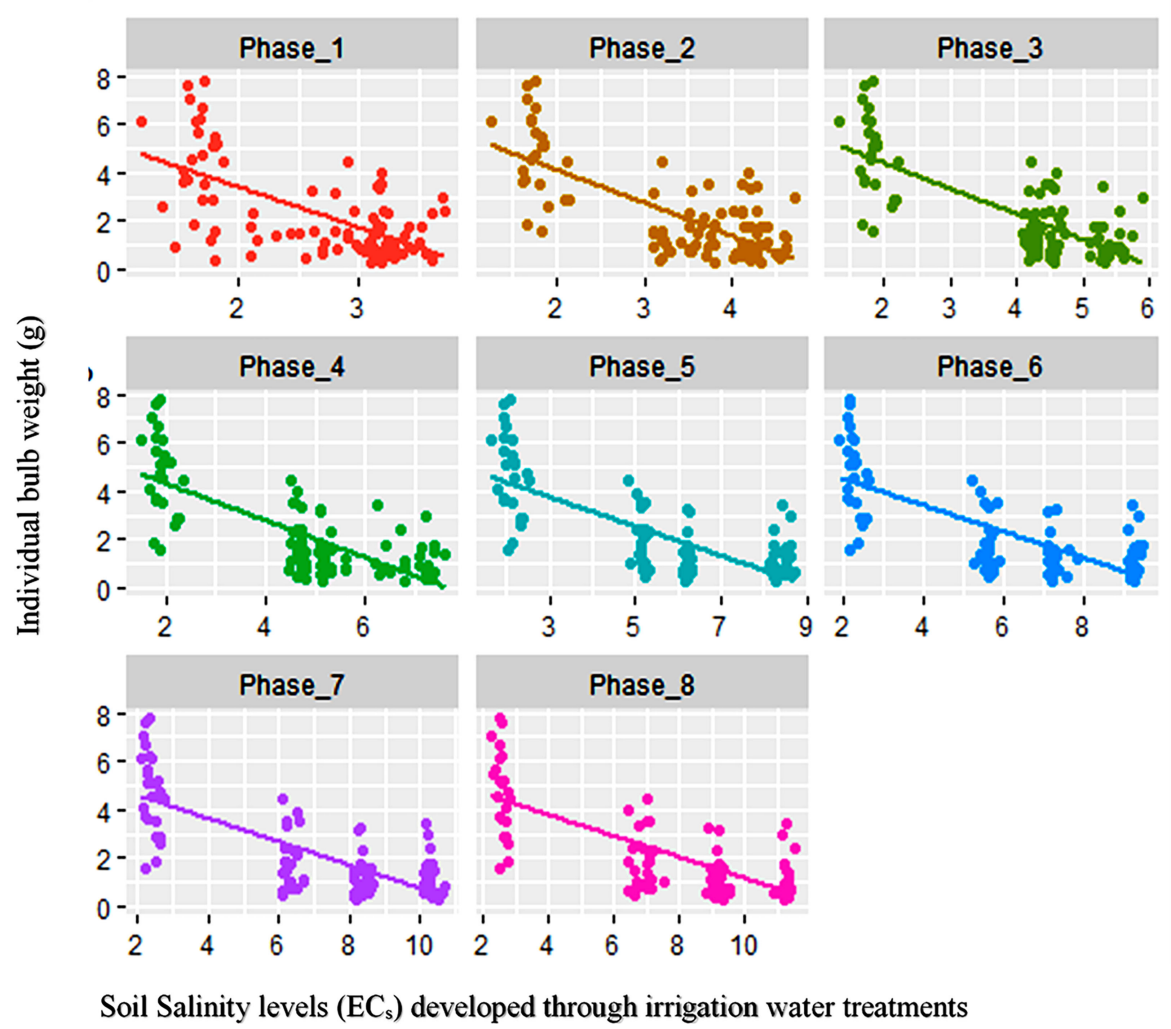
Regression of EC_s_ at different phases on the individual bulb weight of 25 different onion genotypes.

Coefficient of determination (*R* ^2^) values for linear regression varied between 0.39 and 0.58. Multiple regression results revealed that combining all the phases accounted for 61% of the contribution towards the total IBW variation (**Table 8**). **Figure 5** shows the contribution of different phases to the IBW variation. It was clear from the graph that, after phase 4, the attainment of soil salinity level is very distinct.

A further analysis (stepwise regression) was done to find the most critical phases for crop when undergoing salinity stress. Results showed that phases 3, 5, and 7 were responsible for 63% variability and are the most important phases under salinity stress (**Tables 8** and **9**).

**Table 9:** Initial and final model after stepwise regression of different phases of soil salinity levels (EC_s_) on individual bulb weight.

Type	Model
Initial model	IBW ~ Phase_1 + Phase_2 + Phase_3 + Phase_4 + Phase_5 + Phase_6 + Phase_7 + Phase_8
Final model	IBW ~ Phase_3 + Phase_5 + Phase_7

IBW, individual bulb weight; different phases representing the EC_s_ values of all the experimental pots at different phases of the crop cycle.

## 4 Discussion

Salinity is a major environmental stressor that reduces agricultural production and sustainability in arid and semiarid settings by delaying the commencement of germination and subsequent seedling establishment (Uçarlı, 2021). Worldwide, salt has a negative impact on agricultural yield. Approximately 30 agricultural plants currently provide 90% of plant-based human food, and the bulk of these crops, known as glycophytes, are neither salt-tolerant nor salt-sensitive. Because of salt sensitivity, glycophytes make up the majority of cultivated plants. Osmotic stress, ion toxicity, and oxidative stress all have an impact on seed germination and seedling establishment. The negative influence of abiotic stresses, such as salt, heat, and drought, has an undesirable impact on seed germination (Wahid et al., 2007). Inhibition of seed germination, fall in germination percentage, and germination delay are the initial outcomes of salinity (Uçarlı, 2021) and are caused by altering the levels of seed germination stimulants (i.e., Gibberellic Acids (GAs), Abscisic Acid (ABA), membrane permeability, and water behavior) in the seed (Uçarlı, 2021).

Results of the year I study stated that germination percentage was hampered by the different salinity treatments. Germination percentage decreased with the increase in salt concentration. Seed germination primarily increases at low concentrations of salt (NaCl), but at rising concentrations, it was significantly reduced (Abdel-Fattah et al., 1972; Sudha and Riazunnisa, 2015). A similar scenario was also observed in different types of salt solutions (NaCl, CaCl_2_, and MgCl_2_) (Malik and Singh, 1977). Therefore, the selection of genotypes based on salinity stress performance would produce better results (Regessa et al., 2010; Regessa et al., 2022). Though the germination percentage was negatively affected by salt treatment, the impact was not so detrimental for this particular trait as quite a few genotypes showed better performance in terms of germinability. In contrast, many of the genotypes were greatly influenced, showing subsequent growth repression and ultimately dying under different salt treatments. Very high growth repression was observed at elevated salinity treatments for different genotypes that previously showed better germination percentage under an ideal environment. As a whole, the germination process (emergence to first leaf development) is vulnerable to salt stress. This harmful effect may be prompted by Na and Cl ions’ direct influence on embryo viability (Jahromi et al., 2008; Daszkowska-Golec, 2011) or indirectly by decreasing the water availability around seeds (Koyro et al., 2008).

After each irrigation treatment, plants turn brown and ultimately older leaves tend to dry. Sometimes, all the leaves in plants of a few genotypes become completely dry within a week of saline water application. Thus, onion plants’ growth in terms of the number of dried plants, number of green plants, number of total leaves, and MLL was severely affected by salinity stress. The interplay between saline treatments and plant age would result in significant changes in onion leaves, especially in terms of weight (Sta-Baba et al., 2005; Sta-Baba et al., 2010), which might be due to the changes in cells’ osmotic potential following water intake reduction (El-Hendawy et al., 2019). Although many of the dried plants started to produce new leaves after the older leaves became dry, in this process, plant growth was hampered and could not contribute to accumulating the assimilates towards the bulb formation and subsequent development. However, practically robust foliage during the vegetative stage is a must for maximum production (Bosch Serra and Casanova, 2000; Sta-Baba et al., 2005). Similar to this (Bernstein and Ayers, 1953), 50% reduced growth in onion in field plots in Riverside, CA, at an EC value of 4.1 dS m^−1^, was also observed. In the present study, the number of dried plants increased with the increase in salinity levels. Drying out or death of seedlings due to exposure to the shallow root system of onion at high salt concentration (Sta-Baba et al., 2005) was also reported under salt water irrigation in a previous study conducted by De Malach et al. (1989). In contrast, the number of green plants was reduced with the elevated salinity level. Similar to this, the number of total leaves also decreased with the increasing salinity treatments. A reduction in leaf numbers by up to 50% was reported after 45 days in onion in an earlier study (Sta-Baba et al., 2010). Apart from this, it was also revealed that MLL was reduced with the increase in salinity levels. This indicates that salt stress has a negative impact on MLL, i.e., the height of onion plants, and approximately 21% reduction in height was recorded due to this abiotic stress (Sta-Baba et al., 2010). Some of the genotypes showed better leaf length despite the elevated salinity level, which indicates that there was some sort of tolerance mechanism. Those plants had the capability to grow under higher salt stress conditions. The height of onion plants was also studied by different research groups in previous studies, and they reported that plant height reduced as salt levels rose (Janki et al., 2020). Interestingly, many research findings (Bernstein and Hayward, 1958; Bernstein, 1962; Allison, 1964) reported that on saline soils, plant height can be limited or completely inhibited by the following factors: (a) the osmotic effect on plant roots, (b) the toxic effect of accumulated ions in plant tissues, (c) the specific effect of constituent ions, or (d) a combination of all three. Reduction in onion’s vegetative growth and development in terms of plant height, number of green leaves, leaf length, etc. was also reported in earlier findings (Hanci and Cebeci, 2018; Shoaib et al., 2018; Regessa et al., 2022; Sanwal et al., 2022).

Both varietal potential and salt concentration had an extensive effect on different growth and yield attributes of onion (Janki et al., 2020). Onion is very much vulnerable to salt stress (Mangal et al., 1989) with a low EC threshold (Maas and Hoffman, 1977). In the present study, traits related to bulb size and weight were observed under different salinity levels. Differences in the performances of the studied genotypes were recorded at different salt stresses. An increase in BL along with a decrease in BD as well as bulb weight was observed with the increase in salt concentration of irrigation water. A similar finding of hampered onion bulb firmness and size was reported in an earlier study (Venâncio et al., 2022). It denotes that bulb development or growth is adversely influenced by the salt stress accompanied by soil water unavailability triggered by salt concentration. Bulb growth is the most vulnerable (Kadayifci et al., 2005) and tends to escape the stress by reducing the duration of different stages, leading to a faster bulb growth stage under salinized soil (Kamran et al., 2019; Paudel et al., 2020), and ultimately ends with small-sized bulbs, which was common in previous studies. In a previous study (Ayers and Westcot, 1985), it was concluded that onion yield potential is 100% for EC_w_ = 0.8 dS m^−1^, 90% for 1.2 dS m^−1^, 75% for 1.8 dS m^−1^, 50% for 2.9 dS m^−1^, and 0% for 5.0 dS m^−1^. The reduction in yield might be due to fewer bulbs per unit area, as well as reduced bulb size (Sta-Baba et al., 2005). A considerable decrease in fresh bulb weight and bulb volume was associated with the increasing salt concentration (Janki et al., 2020; Regessa et al., 2022; Sanwal et al., 2022; Venâncio et al., 2022). Fifty percent and 80% bulb loss were reported at 3.7 and 9.51 dS m^−1^ solution plots, respectively, compared to the control plot in an earlier study (Sta-Baba et al., 2010).

By delaying the start of germination and subsequent seedling establishment, salinity becomes a significant environmental constraint that lowers agricultural production and stability in arid and semiarid situations. When salt (NaCl) content is low, seed germination is predominantly increased, but as the concentration rises, it is greatly decreased. Onion leaves grow and develop differently depending on plant age and saline treatments. In this investigation, the number of dried plants increased as salinity levels rose. When the salt concentration was higher, the growth rate was significantly slower. MLL, or the height of onion plants, is negatively impacted by salinity exertion. Onion growth and yield characteristics varied greatly depending on both varietal potential and salt content (Hanci and Cebeci, 2018; Shoaib et al., 2018; Regessa et al., 2022; Sanwal et al., 2022). Onion has a low EC threshold, making it extremely susceptible to salt stress. Bulb growth is most susceptible to salinity stress and tends to avoid it by shortening the time between stages.

Soil salinity levels gradually increase with the saline water application as irrigation (Venâncio et al., 2022). The progression increased after consecutive applications up to the fourth irrigation for all the levels and reached the highest point at the end of the crop season. At this point, soil salinity (EC_s_) was almost nearer to the corresponding levels of irrigation water solutions (EC_w_). Similar to this, the gradual evolution pattern and the highest levels of EC_s_ nearer to EC_w_ at the end of the onion crop cycle were also concluded in a study (Sta-Baba et al., 2010).

Stress-associated indices help to select superior genotypes having tolerance to particular stresses. Ranking depending on the stress index values makes it easier to identify the best-performing genotypes. Three different indices were estimated in the current study consisting of indicators based on shoot growth (ShTI), bulb weight (STI), and bulb weight loss under stress (PYR). ShTI recognizes the genotypes that show better shoot growth under salinity stress, while STI could differentiate the better genotypes that have the potential of producing sizable onion bulbs under a salinity stress environment. On the other hand, PYR identifies the genotypes that show minimum bulb yield loss under stress conditions. Results of the present study revealed that the ranking of ShTI, STI, and PYR at different salinity levels showed a dissimilar genotype at the upper positions. For example, Ac Bog 415, Ac Bog 425, and Ac Bog 414 were at the top of the position (rank 1) under salinity levels 8, 10, and 12 dS m^−1^, respectively, for the stress index ShTI. A similar pattern was also observed for the other two indices (STI and PYR). Thus, a combined rank position, including the stress indices and the rank of IBW, was intended to be estimated. In doing so, mean rank (MR) was calculated for all the indices, and subsequently, a fresh ranking was employed based on the mean ranking of indices. To identify the best genotypes, presence in the top 10 ranking in multiple categories (i.e., the rank of IBW and MRs of indices) was counted. The maximum occurring genotypes were believed to have better vegetative growth, yielding ability, and minimum yield loss and ultimately had some sort of tolerance mechanism under salt stress conditions. In the present study, nine genotypes that had those characteristic features were recorded. Genotype performance under stress and sustainable selection would result depending on this (Venâncio et al., 2022).

The result from the regression analysis indicated the significant effects of saline water irrigation towards the bulb weight variation. Corresponding to water salinity (EC_w_), soil salinity had a similar contribution to the variation in IBW. Elevated salinity levels are directly associated with impaired soil nutrient availability, which hampers not only nutrient absorption but also water uptake. Plants under salt stress were forced to undergo physiological changes to combat unfavorable conditions. All these lead to the reduction in onion bulb yield under such stress conditions. Simple as well as multiple linear regressions yielded similar results of soil salinity impact on the IBW. Soil salinity levels of all the phases individually and also combined significantly contributed to the yield variation. A report from a regression analysis on onion bulb yield was found to be affected by stress during the growth stage (Lee et al., 2019). The result of stepwise regression indicated that the middle phases of the growth cycle are the vulnerable stage under salinity stress (phases 3 to 7). This may be due to the fact that at the early vegetative stage, crop plants can recover quickly, while at the last stage, salinity level had very minimal effects, if any, on the crop as, at this point, bulb formation had already been completed. Stepwise regression was employed to account for the variability contributed by predictor variables on onion bulb yield in the previous study and identified the most responsible traits related to the early growth stage (Sanwal et al., 2022). From the regression graph, it was also evident that salinity development in soil due to the saline water was more distinct after consecutive applications of few irrigations (fourth in the case of the present study).

## 6 Conclusions

Onion is one of the most sensitive crops to salt stress as compared to other spice crops. Starting from germination to bulb maturity, all the stages are vulnerable to salinity stress. Reduction in germination percentage along with repression in subsequent growth indicated the adverse influence of higher salt concentration towards early plant formation. Interruption in leaf growth due to stress ensured limited or no food production as well as successive translocation to the bulb, which ultimately ends with reduced production. A higher salt concentration during the bulb formation stage interferes with the bulb development process, resulting in a reduced and irregular bulb shape, volume, and weight of onion bulbs. All these ultimately lead to a reduction in bulb fresh yield. The significant reduction in bulb weight compared to the control treatment clearly defines the detrimental effect of salt stress on onion yield. The variable performance of the studied genotypes under stress conditions specifies the availability of variability among the germplasm for salinity tolerance. The better sustainability and the subsequent bulb formation of several salinity-tolerant genotypes can be used in further research and for cultivation in saline-prone areas. The present findings will improve the current understanding of the salinity tolerance of onion. The present output allows the scope of the developing gene pool to have certain characteristics associated with tolerance to salt stress. Further advanced research related to genomic-level studies can dissect the underlying molecular mechanism behind the salinity tolerance of onion.

## Data availability statement

The original contributions presented in the study are included in the article/**Supplementary Material**, further inquiries can be directed to the corresponding author/s.

## Author contributions

Conceptualization: MA, MAR, MMR, MH, MN, AF, MM, SR, MI, and SM; methodology: MA, MAR, MMR, MH, MN, AF, MM, SR, MI, and SM; validation: MA, MAR, MMR, MH, MN, AF, MM, SR, MI, and SM; formal analysis: MA, AG, AA, and AH; investigation: MA and MAR; resources: MA and AH; data curation: MA, AG, AA, and AH; writing—original draft preparation: MA, MAR, MMR, MH, MN, AF, MM, SR, MI, and SM; editing: AG, AH, and AA; visualization: MA, MAR, MMR, MH, MN, AF, MM, SR, MI, and SM; supervision: AH and MA; funding: AA, AG, SM, and AH. All authors contributed to the article and approved the submitted version.
