# Supplementary material for: Performance valuation of onion (Allium cepa L.) genotypes under different levels of salinity for the development of cultivars suitable for saline regions
Source: Front Plant Sci. 2023 Mar 31;14:1154051. doi: 10.3389/fpls.2023.1154051 (PMC10102481; doi:10.3389/fpls.2023.1154051)
Supplement: Supplementary file 1 [file Table_1.docx]

**Supplementary Tables**

**Table S1.** Details of the onion genotypes used in the present study

| Entry No. | Genotypes | | Source of collection | | Bulb shape | Bulb color |
| --- | --- | --- | --- | --- | --- | --- |
| 1 | Ac Bog 409 | | Spices Research Centre, BARI, Bogura | | Rhomboid | Light Brown |
| 2 | Ac Bog 410 | | -Do- | | Flat globe | Light Brown |
| 3 | Ac Bog 411 | | -Do- | | Globe | Light Brown |
| 4 | Ac Bog 412 | | -Do- | | Broad elliptic | Red |
| 5 | Ac Bog 413 | | -Do- | | Globe | Light Brown |
| 6 | Ac Bog 414 | | -Do- | | Rhomboid | Light Red |
| 7 | Ac Bog 415 | | -Do- | | Rhomboid | Light Brown |
| 8 | Ac Bog 416 | | -Do- | | Ovate | Light Brown |
| 9 | Ac Bog 417 | | -Do- | | Rhomboid | Light Red |
| 10 | Ac Bog 419 | | -Do- | | Flat globe | Light Brown |
| 11 | Ac Bog 420 | | -Do- | | Globe | Light Brown |
| 12 | Ac Bog 421 | | -Do- | | Flat globe | Light Brown |
| 13 | Ac Bog 422 | | -Do- | | Flat globe | Light Brown |
| 14 | Ac Bog 423 | | -Do- | | Flat globe | Light Brown |
| 15 | Ac Bog 424 | | -Do- | | Flat globe | Light Brown |
| 16 | Ac Bog 425 | | -Do- | | Flat globe | Light Brown |
| 17 | Ac Bog 426 | | -Do- | | Flat globe | Light Brown |
| 18 | Ac Bog 427 | | -Do- | | Ovate | Light Red |
| 19 | Ac Bog 428 | | -Do- | | Flat globe | Light Brown |
| 20 | Ac Bog 429 | | -Do- | | Ovate | Light Brown |
| 21 | Ac Bog 430 | | -Do- | | Flat globe | Light Brown |
| 22 | Ac Bog 431 | | -Do- | | Flat globe | Red |
| 23 | Ac Bog 432 | | -Do- | | Globe | Light Brown |
| 24 | Ac Gaz 379 | | Regional Spices Research Centre, BARI, Gazipur | | Flat globe | Red |
| 25 | BARI Piaz-4 | | Spices Research Centre, BARI, Bogura | | Globe | Red |
|  | |  | |  |  |  |

**Table S2.** Average monthly weather data of the experimental period at SRC, BARI, Bogura from November, 2020 to May, 2021

| Month | Temperature (°C) | | Relative humidity (%) | Rainfall (mm) | Rainy day |
| --- | --- | --- | --- | --- | --- |
|  | Min | Max |  |  |  |
| November, 2020 | 21.89 | 30.56 | 68.07 | 0.00 | 0 |
| December, 2020 | 13.98 | 24.88 | 76.19 | 0.00 | 0 |
| January, 2021 | 12.65 | 23.82 | 72.05 | 0.00 | 0 |
| February, 2021 | 15.06 | 29.10 | 51.75 | 0.00 | 0 |
| March, 2021 | 21.26 | 32.74 | 48.71 | 0.19 | 1 |
| April, 2021 | 24.05 | 34.91 | 52.94 | 1.60 | 6 |

Min=Minimum; Max=Maximum

**Table S3.** Analysis of variance of different traits of onion genotypes studied under different salinity stress during second year

| SV | DF | PPDL | PGP | NL | MLL | BL | BD | IBW |
| --- | --- | --- | --- | --- | --- | --- | --- | --- |
| Rep | 1 | 214.2 | 141.2 | 1.93 | 46.13 | 829.77 | 863.69 | 78.11 |
| Genotype (σ^2^g) | 24 | 2156.4** | 1811.2** | 7.09** | 68.46** | 103.65** | 37.71** | 4.264** |
| Salinity level (σ^2^s) | 3 | 13569.9** | 19685.7** | 4.58** | 532.95** | 367.46** | 55.75** | 147.11** |
| Genotype: Salinity level (σ^2^gxs) | 72 | 676.3** | 618.9** | 1.83** | 21.91 | 17.6** | 15.2 | 2.22** |
| Residuals | 89 | 173.8 | 169.7 | 0.87 | 15.74 | 9.85 | 12.77 | 1.17 |

SV= Source of variation; DF= Degrees of freedom; PPDL=Percent plant with dead leaves; PGP= Percent green plants; NL=Number of leaves; MLL=Maximum leaf length; BL=Bulb length; BD=Bulb diameter; IBW=Individual bulb weight; Rep=Replication

** Significant at 1% level of probability

**Table S4.** Influence of different salt concentrations on traits overall mean of onion genotypes

| Item | PPDL | PGP | NL | MLL | BL | BD | IBW |
| --- | --- | --- | --- | --- | --- | --- | --- |
| C (0 dSm^-1^) | 0.04 | 87.00 | 4.79 | 22.69 | 11.66 | 10.42 | 4.75 |
| 8 dSm^-1^ | 0.88 | 54.84 | 4.30 | 17.17 | 18.07 | 11.32 | 1.91 |
| 10 dSm^-1^ | 0.98 | 56.68 | 4.58 | 20.72 | 16.34 | 8.77 | 1.19 |
| 12 dSm^-1^ | 1.36 | 39.62 | 4.11 | 15.51 | 15.10 | 10.20 | 1.10 |
| CV% | 52.69 | 21.88 | 20.97 | 20.76 | 20.52 | 35.11 | 48.23 |
| lsd | 0.17 | 5.17 | 0.37 | - | 1.25 | 1.42 | 0.43 |

PPDL=Percent plant with dead leaves; PGP= Percent green plants; NL=Number of leaves; MLL=Maximum leaf length; BL=Bulb length; BD=Bulb diameter; IBW=Individual bulb weight; C=Control treatment 0 dSm^-1^; CV= Coefficient of variation; lsd= Least significant difference

**Table S5.** Soil salinity level developed at experimental plot throughout crop cycle at 0 dSm^-1^ irrigation water treatments

| Genotype | Phase 1 | Phase 2 | Phase 3 | Phase 4 | Phase 5 | Phase 6 | Phase 7 | Phase 8 |
| --- | --- | --- | --- | --- | --- | --- | --- | --- |
| Ac Bog 409 | 1.88 | 2.11 | 2.23 | 2.37 | 2.51 | 2.63 | 2.73 | 2.87 |
| Ac Bog 410 | 1.79 | 2.09 | 2.17 | 2.21 | 2.31 | 2.55 | 2.65 | 2.71 |
| Ac Bog 411 | 1.71 | 2.15 | 2.19 | 2.25 | 2.35 | 2.41 | 2.51 | 2.63 |
| Ac Bog 412 | 1.37 | 1.91 | 2.11 | 2.17 | 2.31 | 2.45 | 2.63 | 2.75 |
| Ac Bog 414 | 1.81 | 1.85 | 1.91 | 2.11 | 2.19 | 2.31 | 2.55 | 2.65 |
| Ac Bog 415 | 1.65 | 1.71 | 1.79 | 1.91 | 2.11 | 2.26 | 2.41 | 2.55 |
| Ac Bog 416 | 1.8 | 1.85 | 1.89 | 1.93 | 2.15 | 2.22 | 2.45 | 2.59 |
| Ac Bog 417 | 1.81 | 1.83 | 1.86 | 1.95 | 2.11 | 2.22 | 2.29 | 2.35 |
| Ac Bog 413 | 1.61 | 1.71 | 1.81 | 1.93 | 2.17 | 2.25 | 2.37 | 2.45 |
| Ac Bog 419 | 1.73 | 1.75 | 1.83 | 1.88 | 2.09 | 2.15 | 2.31 | 2.49 |
| Ac Bog 420 | 1.83 | 1.85 | 1.88 | 1.93 | 2.11 | 2.25 | 2.31 | 2.55 |
| Ac Bog 421 | 1.59 | 1.63 | 1.71 | 1.81 | 1.91 | 2.11 | 2.22 | 2.51 |
| Ac Bog 422 | 1.69 | 1.71 | 1.76 | 1.79 | 1.93 | 2.17 | 2.31 | 2.56 |
| Ac Bog 423 | 1.58 | 1.65 | 1.71 | 1.81 | 1.93 | 2.11 | 2.19 | 2.59 |
| Ac Bog 424 | 1.67 | 1.75 | 1.77 | 1.81 | 1.91 | 2.09 | 2.25 | 2.37 |
| Ac Bog 425 | 1.71 | 1.76 | 1.79 | 1.83 | 1.98 | 2.13 | 2.21 | 2.53 |
| Ac Bog 426 | 1.61 | 1.65 | 1.69 | 1.73 | 1.95 | 2.11 | 2.18 | 2.25 |
| Ac Bog 427 | 1.55 | 1.61 | 1.71 | 1.81 | 1.93 | 2.15 | 2.28 | 2.56 |
| Ac Bog 428 | 1.81 | 1.83 | 1.85 | 1.89 | 1.96 | 2.11 | 2.3 | 2.59 |
| Ac Bog 429 | 1.56 | 1.61 | 1.65 | 1.69 | 1.79 | 2.08 | 2.18 | 2.73 |
| Ac Bog 430 | 1.21 | 1.25 | 1.35 | 1.51 | 1.63 | 1.9 | 2.11 | 2.51 |
| Ac Bog 431 | 1.73 | 1.81 | 1.88 | 1.93 | 2.15 | 2.31 | 2.51 | 2.71 |
| Ac Bog 432 | 1.81 | 1.83 | 1.85 | 1.89 | 2.01 | 2.11 | 2.23 | 2.53 |
| Ac Gaz 379 | 1.71 | 1.75 | 1.79 | 1.88 | 2.45 | 2.55 | 2.61 | 2.75 |
| BARI Piaz-4 | 1.63 | 1.67 | 1.69 | 1.75 | 2.11 | 2.35 | 2.51 | 2.75 |
| Mean | 1.67 | 1.77 | 1.83 | 1.91 | 2.08 | 2.24 | 2.37 | 2.58 |
| SD | 0.15 | 0.18 | 0.19 | 0.19 | 0.2 | 0.17 | 0.17 | 0.14 |
| Min | 1.21 | 1.25 | 1.35 | 1.51 | 1.63 | 1.9 | 2.11 | 2.25 |
| Max | 1.88 | 2.15 | 2.23 | 2.37 | 2.51 | 2.63 | 2.73 | 2.87 |

SD= Standard deviation; Min= Minimum; Max= Maximum

**Table S6.** Soil salinity level developed at experimental plot throughout crop cycle at 8 dSm^-1^ irrigation water treatments

| Genotype | Phase 1 | Phase 2 | Phase 3 | Phase 4 | Phase 5 | Phase 6 | Phase 7 | Phase 8 |
| --- | --- | --- | --- | --- | --- | --- | --- | --- |
| Ac Bog 409 | 2.91 | 3.21 | 4.23 | 4.51 | 4.81 | 5.17 | 6.11 | 7.04 |
| Ac Bog 410 | 3.1 | 3.59 | 4.61 | 4.71 | 4.89 | 5.23 | 6.29 | 6.71 |
| Ac Bog 411 | 2.45 | 3.61 | 4.71 | 4.81 | 5.11 | 5.61 | 6.23 | 6.64 |
| Ac Bog 412 | 2.98 | 3.24 | 4.63 | 4.75 | 5.21 | 5.71 | 6.31 | 7.01 |
| Ac Bog 414 | 3.15 | 3.65 | 4.65 | 4.72 | 5.11 | 5.51 | 6.13 | 7.11 |
| Ac Bog 415 | 3.21 | 4.01 | 4.51 | 4.63 | 5.04 | 5.62 | 6.31 | 6.54 |
| Ac Bog 416 | 3.61 | 4.11 | 4.29 | 4.55 | 5.11 | 5.73 | 6.41 | 7.05 |
| Ac Bog 417 | 3.17 | 4.21 | 4.56 | 4.71 | 5.12 | 5.61 | 6.21 | 6.79 |
| Ac Bog 413 | 3.19 | 4.19 | 4.61 | 4.64 | 5.05 | 5.41 | 6.51 | 6.46 |
| Ac Bog 419 | 3.08 | 4.11 | 4.53 | 4.67 | 5.13 | 5.51 | 6.69 | 7.13 |
| Ac Bog 420 | 3.11 | 3.75 | 4.51 | 4.62 | 5.06 | 5.41 | 6.11 | 7.05 |
| Ac Bog 421 | 3.23 | 3.81 | 4.63 | 4.81 | 5.21 | 5.61 | 6.71 | 7.51 |
| Ac Bog 422 | 3.12 | 3.69 | 4.62 | 4.71 | 5.23 | 5.64 | 6.51 | 7.11 |
| Ac Bog 423 | 3.51 | 3.41 | 4.11 | 4.52 | 5.18 | 5.89 | 6.72 | 7.15 |
| Ac Bog 424 | 3.29 | 4.01 | 4.56 | 4.63 | 5.21 | 5.61 | 6.11 | 6.62 |
| Ac Bog 425 | 2.96 | 4.12 | 4.69 | 4.74 | 5.23 | 5.63 | 6.19 | 6.89 |
| Ac Bog 426 | 3.07 | 4.14 | 4.52 | 4.81 | 5.31 | 5.67 | 6.22 | 6.72 |
| Ac Bog 427 | 3.16 | 4.11 | 4.44 | 4.53 | 5.13 | 5.51 | 6.24 | 7.02 |
| Ac Bog 428 | 3.19 | 4.15 | 4.37 | 4.61 | 5.12 | 5.61 | 6.31 | 7.04 |
| Ac Bog 429 | 3.23 | 3.87 | 4.41 | 4.63 | 5.33 | 5.71 | 6.51 | 7.22 |
| Ac Bog 430 | 3.18 | 3.73 | 4.52 | 4.61 | 5.21 | 5.81 | 6.61 | 7.11 |
| Ac Bog 431 | 3.15 | 3.81 | 4.29 | 4.51 | 5.12 | 5.61 | 6.21 | 6.41 |
| Ac Bog 432 | 3.21 | 3.29 | 4.24 | 4.47 | 5.19 | 5.52 | 6.51 | 7.18 |
| Ac Gaz 379 | 3.13 | 4.05 | 4.51 | 4.71 | 5.21 | 5.72 | 6.11 | 6.41 |
| BARI Piaz-4 | 3.05 | 4.16 | 4.61 | 4.81 | 5.21 | 5.71 | 6.41 | 7.01 |
| Mean | 3.14 | 3.84 | 4.49 | 4.66 | 5.14 | 5.59 | 6.35 | 6.92 |
| SD | 0.21 | 0.32 | 0.16 | 0.1 | 0.11 | 0.16 | 0.2 | 0.28 |
| Min | 2.45 | 3.21 | 4.11 | 4.47 | 4.81 | 5.17 | 6.11 | 6.41 |
| Max | 3.61 | 4.21 | 4.71 | 4.81 | 5.33 | 5.89 | 6.72 | 7.51 |

SD= Standard deviation; Min= Minimum; Max= Maximum

**Table S7.** Soil salinity level developed at experimental plot throughout crop cycle at 10 dSm^-1^ irrigation water treatments

| Genotype | Phase 1 | Phase 2 | Phase 3 | Phase 4 | Phase 5 | Phase 6 | Phase 7 | Phase 8 |
| --- | --- | --- | --- | --- | --- | --- | --- | --- |
| Ac Bog 409 | 2.11 | 3.51 | 4.21 | 5.09 | 6.21 | 7.11 | 8.28 | 9.19 |
| Ac Bog 410 | 1.48 | 3.11 | 4.31 | 4.71 | 6.15 | 7.21 | 8.31 | 9.21 |
| Ac Bog 411 | 2.11 | 3.71 | 4.56 | 5.11 | 6.23 | 7.11 | 8.56 | 8.81 |
| Ac Bog 412 | 1.78 | 3.21 | 4.31 | 5.61 | 6.31 | 7.23 | 8.11 | 9.23 |
| Ac Bog 414 | 1.81 | 3.17 | 4.19 | 4.81 | 6.19 | 7.21 | 8.14 | 9.11 |
| Ac Bog 415 | 2.12 | 3.51 | 4.11 | 5.31 | 6.21 | 7.13 | 8.36 | 9.15 |
| Ac Bog 416 | 2.31 | 3.21 | 4.21 | 5.12 | 6.28 | 7.28 | 8.51 | 9.12 |
| Ac Bog 417 | 2.71 | 4.51 | 4.32 | 5.31 | 6.31 | 7.13 | 8.31 | 9.11 |
| Ac Bog 413 | 2.17 | 3.21 | 4.31 | 5.18 | 6.28 | 7.81 | 8.56 | 9.26 |
| Ac Bog 419 | 2.51 | 3.11 | 4.11 | 5.19 | 6.11 | 7.11 | 8.33 | 8.98 |
| Ac Bog 420 | 2.61 | 3.53 | 4.18 | 5.11 | 6.21 | 7.31 | 8.31 | 8.89 |
| Ac Bog 421 | 2.72 | 3.41 | 4.22 | 5.19 | 6.19 | 7.11 | 8.15 | 8.96 |
| Ac Bog 422 | 2.63 | 3.21 | 4.34 | 5.31 | 6.28 | 7.25 | 8.51 | 8.92 |
| Ac Bog 423 | 2.83 | 3.27 | 4.21 | 5.61 | 6.31 | 7.28 | 8.23 | 9.21 |
| Ac Bog 424 | 2.81 | 3.11 | 4.33 | 5.12 | 6.27 | 7.11 | 8.25 | 9.23 |
| Ac Bog 425 | 2.91 | 3.19 | 4.31 | 5.08 | 6.25 | 7.18 | 8.11 | 9.31 |
| Ac Bog 426 | 3.11 | 3.55 | 4.51 | 5.31 | 6.23 | 7.22 | 8.63 | 9.31 |
| Ac Bog 427 | 3.12 | 3.71 | 4.31 | 5.11 | 6.11 | 7.21 | 8.11 | 9.51 |
| Ac Bog 428 | 3.11 | 3.81 | 4.56 | 5.13 | 6.18 | 7.18 | 8.21 | 9.32 |
| Ac Bog 429 | 3.01 | 3.51 | 4.33 | 5.32 | 6.16 | 7.29 | 8.55 | 9.23 |
| Ac Bog 430 | 2.81 | 3.11 | 4.35 | 5.13 | 6.25 | 7.61 | 8.61 | 9.31 |
| Ac Bog 431 | 2.69 | 3.51 | 4.22 | 5.21 | 6.11 | 7.36 | 8.33 | 9.51 |
| Ac Bog 432 | 2.56 | 3.21 | 4.51 | 5.14 | 6.15 | 7.56 | 8.39 | 9.31 |
| Ac Gaz 379 | 3.11 | 3.72 | 4.19 | 5.11 | 6.27 | 7.21 | 8.69 | 9.02 |
| BARI Piaz-4 | 3.09 | 3.53 | 4.21 | 5.61 | 6.31 | 7.31 | 8.36 | 9.33 |
| Mean | 2.57 | 3.43 | 4.3 | 5.2 | 6.22 | 7.26 | 8.36 | 9.18 |
| SD | 0.47 | 0.32 | 0.13 | 0.21 | 0.07 | 0.17 | 0.18 | 0.18 |
| Min | 1.48 | 3.11 | 4.11 | 4.71 | 6.11 | 7.11 | 8.11 | 8.81 |
| Max | 3.12 | 4.51 | 4.56 | 5.61 | 6.31 | 7.81 | 8.69 | 9.51 |

SD= Standard deviation; Min= Minimum; Max= Maximum

**Table S8.** Soil salinity level developed at experimental plot throughout crop cycle at 12 dSm^-1^ irrigation water treatments

| Genotype | Phase 1 | Phase 2 | Phase 3 | Phase 4 | Phase 5 | Phase 6 | Phase 7 | Phase 8 |
| --- | --- | --- | --- | --- | --- | --- | --- | --- |
| Ac Bog 409 | 3.19 | 4.21 | 5.28 | 6.41 | 8.24 | 9.48 | 10.22 | 11.18 |
| Ac Bog 410 | 3.22 | 4.31 | 5.39 | 6.21 | 8.11 | 9.18 | 10.31 | 11.31 |
| Ac Bog 411 | 3.24 | 4.28 | 5.11 | 6.31 | 8.21 | 9.14 | 10.11 | 10.91 |
| Ac Bog 412 | 2.91 | 4.61 | 5.25 | 7.11 | 8.61 | 9.23 | 10.18 | 11.31 |
| Ac Bog 414 | 3.61 | 4.21 | 5.28 | 7.21 | 8.28 | 9.13 | 10.33 | 11.32 |
| Ac Bog 415 | 3.71 | 4.29 | 5.54 | 6.71 | 8.21 | 9.11 | 10.31 | 11.51 |
| Ac Bog 416 | 3.46 | 4.61 | 5.31 | 6.81 | 8.17 | 9.19 | 10.16 | 11.23 |
| Ac Bog 417 | 3.16 | 4.28 | 5.37 | 7.28 | 8.11 | 9.28 | 10.25 | 11.39 |
| Ac Bog 413 | 3.19 | 4.22 | 5.31 | 7.31 | 8.25 | 9.13 | 10.18 | 11.11 |
| Ac Bog 419 | 3.15 | 4.29 | 5.55 | 7.41 | 8.39 | 9.22 | 10.39 | 11.27 |
| Ac Bog 420 | 3.15 | 4.28 | 5.31 | 6.23 | 8.41 | 9.18 | 10.14 | 11.25 |
| Ac Bog 421 | 3.23 | 4.41 | 5.11 | 6.78 | 8.51 | 9.11 | 10.27 | 11.33 |
| Ac Bog 422 | 3.51 | 4.33 | 5.17 | 7.13 | 8.53 | 9.23 | 10.14 | 11.19 |
| Ac Bog 423 | 3.59 | 4.11 | 5.39 | 7.22 | 8.55 | 9.22 | 10.61 | 11.12 |
| Ac Bog 424 | 3.69 | 4.71 | 5.91 | 7.21 | 8.61 | 9.37 | 10.23 | 11.16 |
| Ac Bog 425 | 3.37 | 4.61 | 5.75 | 7.61 | 8.22 | 9.39 | 10.12 | 11.31 |
| Ac Bog 426 | 3.27 | 4.19 | 5.63 | 6.38 | 8.33 | 9.36 | 10.23 | 11.33 |
| Ac Bog 427 | 3.36 | 4.51 | 5.61 | 6.51 | 8.31 | 9.31 | 10.61 | 11.37 |
| Ac Bog 428 | 3.18 | 4.33 | 5.31 | 6.79 | 8.29 | 9.25 | 10.51 | 11.22 |
| Ac Bog 429 | 3.29 | 4.21 | 5.55 | 7.27 | 8.51 | 9.26 | 10.31 | 11.33 |
| Ac Bog 430 | 3.41 | 4.39 | 5.31 | 7.39 | 8.61 | 9.33 | 10.36 | 11.22 |
| Ac Bog 431 | 3.55 | 4.51 | 5.59 | 7.37 | 8.71 | 9.18 | 10.33 | 11.36 |
| Ac Bog 432 | 3.21 | 4.55 | 5.61 | 7.34 | 8.23 | 9.23 | 10.37 | 11.14 |
| Ac Gaz 379 | 3.41 | 4.61 | 5.51 | 7.11 | 8.22 | 9.21 | 10.11 | 11.19 |
| BARI Piaz-4 | 3.25 | 4.11 | 5.54 | 6.41 | 8.18 | 9.28 | 10.71 | 11.33 |
| Mean | 3.33 | 4.37 | 5.43 | 6.94 | 8.35 | 9.24 | 10.3 | 11.26 |
| SD | 0.2 | 0.17 | 0.2 | 0.43 | 0.18 | 0.09 | 0.16 | 0.12 |
| Min | 2.91 | 4.11 | 5.11 | 6.21 | 8.11 | 9.11 | 10.11 | 10.91 |
| Max | 3.71 | 4.71 | 5.91 | 7.61 | 8.71 | 9.48 | 10.71 | 11.51 |

SD= Standard deviation; Min= Minimum; Max= Maximum
